# High-Resolution Fluorescence Imaging Combined With Computer Simulations to Quantitate Surface Dynamics and Nanoscale Organization of Neuroligin-1 at Synapses

**DOI:** 10.3389/fnsyn.2022.835427

**Published:** 2022-04-25

**Authors:** Matthieu Lagardère, Adèle Drouet, Matthieu Sainlos, Olivier Thoumine

**Affiliations:** CNRS, Interdisciplinary Institute for Neuroscience, IINS, UMR 5297, University of Bordeaux, Bordeaux, France

**Keywords:** adhesion molecule, membrane diffusion, single molecule tracking, computer simulation, fluorescence recovery after photo bleaching

## Abstract

Neuroligins (NLGNs) form a family of cell adhesion molecules implicated in synapse development, but the mechanisms that retain these proteins at synapses are still incompletely understood. Recent studies indicate that surface-associated NLGN1 is diffusionally trapped at synapses, where it interacts with quasi-static scaffolding elements of the post-synaptic density. Whereas single molecule tracking reveals rapid diffusion and transient immobilization of NLGN1 at synapses within seconds, fluorescence recovery after photobleaching experiments indicate instead a long-term turnover of NLGN1 at synapse, in the hour time range. To gain insight into the mechanisms supporting NLGN1 anchorage at post-synapses and try to reconcile those experimental paradigms, we quantitatively analyzed here live-cell and super-resolution imaging experiments performed on NLGN1 using a newly released simulator of membrane protein dynamics for fluorescence microscopy, FluoSim. Based on a small set of parameters including diffusion coefficients, binding constants, and photophysical rates, the framework describes fairly well the dynamic behavior of extra-synaptic and synaptic NLGN1 over both short and long time ranges, and provides an estimate of NLGN1 copy numbers in post-synaptic densities at steady-state (around 50 dimers). One striking result is that the residence time of NLGN1 at synapses is much longer than what can be expected from extracellular interactions with pre-synaptic neurexins only, suggesting that NLGN1 is stabilized at synapses through multivalent interactions with intracellular post-synaptic scaffolding proteins.

## Introduction

During neuronal development, several adhesion protein families are involved in establishing and maintaining synaptic connections, among which the neurexins (NRXNs) and their binding partners neuroligins (NLGNs) have been widely studied (Bemben et al., 2015; Südhof, 2017). These transmembrane molecules are implicated in a variety of extracellular and intracellular protein-protein interactions, including calcium-dependent *trans*-synaptic binding between NRXN and NLGN ectodomains (Levinson and El-Husseini, 2007), *cis*-interactions with neurexophilin and MDGAs, respectively (Born et al., 2014; Connor et al., 2019), and C-terminal binding to PDZ-domain containing scaffolding proteins such as CASK and PSD-95, respectively (Irie et al., 1997; Mukherjee et al., 2008). NRXNs and NLGNs are involved in regulating synaptic differentiation and potentiation through either direct or indirect connections to pre-synaptic calcium channels and post-synaptic neurotransmitter receptors, respectively (Missler et al., 2003; Poulopoulos et al., 2009; Shipman et al., 2011; Nguyen et al., 2016; Haas et al., 2018; Letellier et al., 2018, 2020; Wu et al., 2019).

The large repertoire of protein interactions displayed by NRXNs and NLGNs allows a fine regulation of the membrane trafficking and synaptic retention of these molecules. Indeed, both NRXNs and NLGNs were shown by single molecule tracking to be highly dynamic in the neuronal plasma membrane, and transiently trapped at synapses through a combination of extracellular and intracellular protein interactions (Neupert et al., 2015; Chamma et al., 2016a; Klatt et al., 2021). Synaptic confinement of these molecules increases as synapses mature during neuronal development (Chamma et al., 2016a), and super-resolution microscopy investigation in mature synapses showed that NRXN and NLGN form small confinement domains facing each other on both sides of the synaptic cleft (Chamma et al., 2016a,b; Trotter et al., 2019). Despite these advances, the molecular mechanisms that regulate the surface dynamics, synaptic anchorage and nanoscale localization of NLGNs, are still unclear. Part of the difficulty in interpreting NLGN surface dynamics or localization data arises from the various imaging techniques used i.e., Single Particule Tracking (SPT), fluorescence recovery after photobleaching (FRAP) and stochastic optical reconstruction microscopy (STORM), which are often performed at different protein expression levels, probe labeling density, and recording time scales.

To address these limitations, we provide here a detailed quantitative description of the membrane dynamics and nanoscale distribution of NLGN1 in neurons, by correlating imaging experiments and computer simulations. We previously applied such a modeling approach to evaluate the mechanisms controlling AMPA receptor trafficking at synapses (Czöndör et al., 2012). In this study, we took advantage of our recently released simulator of membrane protein dynamics, FluoSim, that was thoroughly validated against live-cell and super-resolution imaging experiments performed on lamellipodial contacts mediated by NRXN-NLGN adhesions in heterologous cells (Lagardère et al., 2020). We extended this analysis to model NLGN1 dynamics and organization in the neuronal membrane, with a systematic comparison to single molecule tracking and localization studies, as well as long-term FRAP experiments performed in primary hippocampal neurons. This approach allowed us to unify the different imaging paradigms within a single framework using a small set of parameters, i.e., diffusion coefficients outside and inside synapses, as well as binding and unbinding constants to synaptic scaffolds, and photophysical rates. Overall, we offer a simulation package of NLGN1 dynamics at the single molecule and ensemble levels, that closely matches actual imaging data and can be further used to model other types of experiments and/or to adjust labeling conditions and microscopy settings.

## Results

### Diffusional Trapping of NLGN1 at Synapses

**FIGURE 1:**
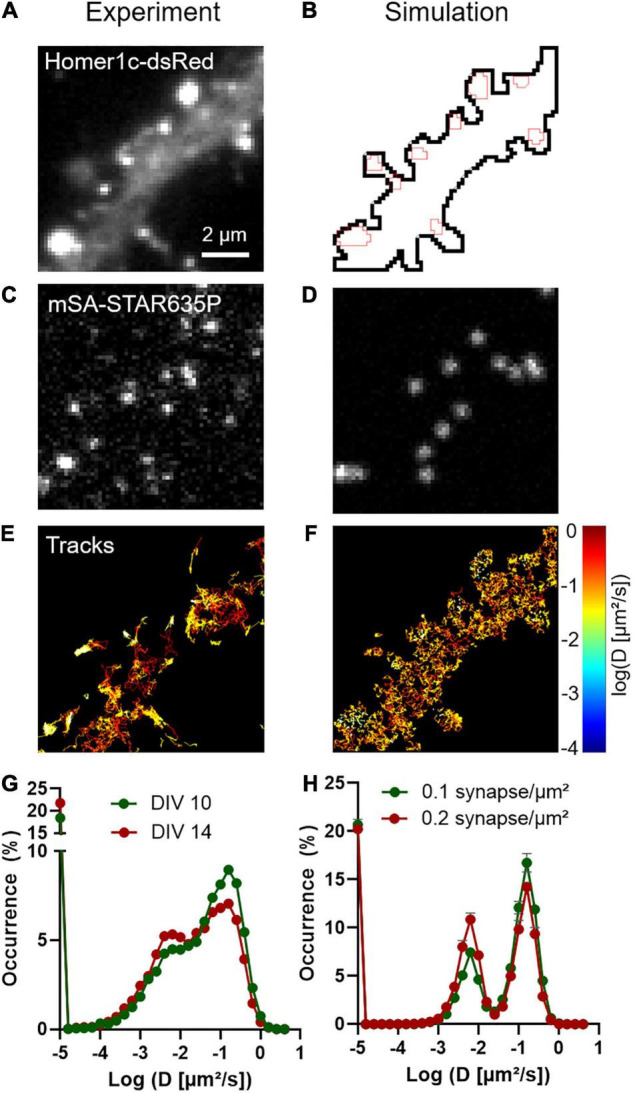
Diffusional trapping of NLGN1 at synapses probed by uPAINT. **(A)** Raw image of a dendritic segment from a neuron co-expressing BFP + shRNA to NLGN1, AP-NLGN1, BirA^ER^, and Homer1c-DsRed. **(B)** The Homer1c-DsRed image was used as a reference to draw the dendrite outline (black) and the PSDs (red areas) entered in the simulator. **(C)** Biotinylated AP-NLGN1 was sparsely labeled with STAR635P-conjugated mSA, allowing the tracking of individual NLGN1 molecules by uPAINT. **(D)** Realistic fluorescence rendering of simulated individual NLGN1 molecules in the defined geometry. **(E)** Image of individual NLGN1 trajectories (2667 tracks), with a color code representing the diffusion coefficient (red = fast diffusion, yellow = slow diffusion). Note the slower NLGN1 diffusion in PSDs. **(F)** Simulated NLGN1 trajectories based on the diffusion coefficients obtained from uPAINT and binding coefficients deduced from FRAP experiments (621 trajectories). The diffusion color code in logarithmic scale applies to both E and F panels. **(G,H)** Semi-log distribution of NLGN1 diffusion coefficients obtained by experiment and simulation, respectively. The experimental data is the average distribution of 13 and 9 neurons at DIV 10 and 14, respectively, the number of trajectories analyzed per cell ranging from 949 to 3113. The simulated data is the average ± sem of 5 independent simulations, with trajectory numbers between 625 and 638 per simulation, generated for two different synapse densities (0.1 and 0.2 synapse/μm^2^, respectively) from the same dendritic geometry (135 μm^2^). The coefficient χ^2^ expressing the goodness of fit between simulated and experimental data was 5.6 and 10.5 for DIV 10 and 14 neurons, respectively, as calculated from 28 binned values.

To characterize NLGN1 dynamics in the dendritic membrane, we first experimentally tracked single recombinant surface NLGN1 molecules in dissociated rat hippocampal neurons using universal Point Acquisition In Nanoscale Topography (uPAINT) (Giannone et al., 2010). To detect NLGN1 at near-endogenous levels we electroporated neurons with shRNA against NLGN1, resulting in a 70% knock-down of native NLGN1 within 2 weeks (Chamma et al., 2016a), and replaced it with a rescue construct bearing a 15-aa N-terminal acceptor peptide (AP) tag which is biotinylated upon the co-expression of the biotin ligase BirA^ER^ (Howarth et al., 2005). Neurons also expressed Homer1c-DsRed as a post-synaptic marker (Kuriu et al., 2006) (Figure 1A). Biotinylated AP-NLGN1 at the cell surface was then detected by sparse labeling with STAR635P-conjugated monomeric streptavidin (mSA) (Demonte et al., 2013; Chamma et al., 2016a), upon oblique illumination from a 647 nm laser (Figure 1C). Single molecule trajectories were reconstructed offline (Figure 1E), and their diffusion coefficient was calculated and plotted on a logarithmic scale. The global distribution of diffusion coefficients for AP-NLGN1 was rather broad, but showed two clear peaks: i) a fast diffusing population (mostly corresponding to extra-synaptic NLGN1 molecules) which peaked at 0.15 μm^2^/s, and a population corresponding to more confined synaptic molecules with diffusion coefficient peaking at 0.006 μm^2^/s) (Figure 1G). When comparing neurons between days *in vitro* (DIV) 10 and 14, the fraction of highly mobile NLGN1 molecules decreased to the benefit of confined molecules, most likely reflecting the formation and/or maturation of synapses that occurs during that time frame (Chanda et al., 2017). Independently of neuronal age, a 20% fraction of immobile molecules was also detected and placed at D = 10^–5^ μm^2^/s. This value is comparable to that obtained with antibodies to AMPA receptors in similar imaging conditions (Nair et al., 2013), and might correspond to a variety of processes, including receptor endocytosis during live labeling, connection to the underlying cytoskeleton, and some degree of non-specific binding of the dye-conjugated probes to the cell surface.

### Introducing Biophysical Parameters in FluoSim

FluoSim is an interactive simulator of membrane protein dynamics for fluorescence live-cell and super-resolution imaging (SRI) techniques (Lagardère et al., 2020). The program calculates in real time the localization and intensity of thousands of independent molecules in 2D cellular geometries, providing simulated data directly comparable to actual experiments. FluoSim requires several inputs: (1) a realistic cellular geometry defined from a microscopy image, comprising potential sub-compartments with specific trapping properties; (2) a given number of molecules that populates the cellular geometry; (3) kinetic parameters (diffusion coefficients, binding and unbinding rates) characterizing the molecular system of interest; and (4) fluorescence photophysical rates related to the experiment to model (Table 1).

**TABLE 1:** Biophysical parameters.

Category	Parameter	Notation	Unit/format	Experiment
Molecules	Copy number*		2,000–20,000	
Times	Length scale of simulations*		2,000–40,000 frames (40–1800 s)	
	Time step*	Δ*t*	20–100 ms	
Diffusion coefficients	Outside synapse	*D*_*out*_	0.15 μm^2^/s	uPAINT
	Inside synapse	*D*_*in*_	0.06 μm^2^/s	Enrichment
	Trapped	*D*_*trap*_	0.006 μm^2^/s	uPAINT
	Crossing probability	*P*_*crossing*_	60%	Enrichment
Kinetics	Binding rate	*k*_*on*_	0.0008 s^–1^	FRAP
	Unbinding rate	*k*_*off*_	0.0005 s^–1^	FRAP
	Immobile fraction		20%	uPAINT
Photophysics	Switch-on rate*	*K_*ON*_*^Fluo^**	0.004–10 s^–1^	uPAINT/STORM
	Switch-off rate*	*K_*OFF*_*^Fluo^**	0–6.4 s^–1^	uPAINT/STORM
	Photobleaching rate*	*K_*OFF*_*^Bleach^**	4 s^–1^	FRAP

*See the methods below for the specific molecule numbers and photo-physical parameters used in the various imaging modes (SPT, FRAP, dSTORM).

To model our experiments in dissociated neurons, we first entered in FluoSim a representative dendritic segment of 48 μm in length, populated by 24 synapses based on the Homer1c-DsRed fluorescence signal (Figure 1B). This represents on average one synapse every 2 μm, as previously reported in DIV 14 hippocampal cultures (Czöndör et al., 2012). In the absence of *a priori* knowledge of the surface density of recombinant NLGN1, we filled the dendritic geometry with an arbitrarily low number of molecules (i.e., 2,500 for a surface area of 70 μm^2^). The synapse is considered as a trapping element for surface diffusing NLGN1 molecules, with excess number of slots based on the large number of scaffolding proteins per post-synaptic density (PSD) (>300 copies) (Chen et al., 2005; Sheng and Hoogenraad, 2007).

Regarding dynamic properties, NLGN1 molecules were allowed to diffuse relatively fast in the dendritic shaft (*D*_out_ = 0.15 μm^2^/s), more slowly in the PSD due to steric hindrance (*D*_in_ = 0.06 μm^2^/s), and very slowly when NLGN1 molecules were considered bound to the PSD (*D*_trap_ = 0.006 μm^2^/s), based on the experimental peak values described above. We also defined a 20% fraction of immobile molecules, as found experimentally, that were placed randomly in the dendritic geometry. To take into account the fact that the synaptic cleft is a narrow cell-cell junction (Tanaka et al., 2012), where large molecules such as NLGN1 can have some difficulty to access, we also introduced in FluoSim a parameter called “crossing probability” (*P*_crossing_ = 0.6) that represents the fraction of molecules allowed to enter the synapse through diffusion, based on our previous estimate of NRXN1β penetration in cell–cell contacts (Lagardère et al., 2020).

To model the transitions between bound and unbound states, we introduced kinetic rates (*k*_on_ and *k*_off_) as global parameters that characterize the dynamic trapping of NLGN1 at synapses through both extracellular and intracellular interactions. The reference values that describe the extracellular interaction between NLGN1 and NRXN1β are in the range of *k*_on_ = 0.15 s^–1^ and *k*_off_ = 0.015 s^–1^ (Comoletti et al., 2003; Saint-Michel et al., 2009; Lagardère et al., 2020). However, to match the FRAP experiments performed on AP-NLGN1 or NLGN1-GFP at synapses (Chamma et al., 2016a) (also see below), we had to choose interaction rates 30–150 fold lower (*k*_on_ = 0,0008 s^–1^ and *k*_off_ = 0.0005 s^–1^). This finding indicates that NLGN1 is not solely retained at synapses through its *trans*-synaptic binding to NRXNs, but also forms long-lived bonds with the post-synaptic scaffold, and that the combination of these extracellular and intracellular interactions overall contributes to very low kinetic rates. The molecular enrichment, defined as the ratio between NLGN1 accumulated at PSDs versus NLGN1 present in the shaft, is theoretically given by the formula (*P_*crossing*_ D_*out*/_D_*in*_*) (1 + *k*_*on*_/*k*_*off*_) (Lagardère et al., 2020), and is slightly lowered by the presence of immobile NLGN1 that are placed randomly. Given the chosen parameters, the synaptic enrichment of NLGN1 is predicted to be around 3.5, close to values measured earlier (Chamma et al., 2016a; Toledo et al., 2022).

With respect to photophysical parameters, we defined a fluorescence switch-on rate (*k*_ON_*^Fluo^* = 0.03 s^–1^) that mimics the stochastic binding of the mSA probe in uPAINT so as to match the average surface density of emitting fluorophores (0.6/μm^2^) per time frame (Δt = 20 ms), which is considered as constant. Note that in the absence of knowledge about the actual number of NLGN1 molecules in the neuronal membrane (see direct stochastic optical reconstruction microscopy (dSTORM) experiments below), this value is somewhat arbitrary since the parameter *k*_ON_^Fluo^ is inversely related to the surface density of molecules introduced in the geometry, i.e., if we placed more molecules we would have to choose a lower *k*_ON_^Fluo^ and vice versa. Besides, we set a fluorescence switch-off rate (*k*_OFF_^Fluo^ = 5.4 s^–1^) characterizing the photobleaching rate of the dye STAR635P in the experimental laser excitation conditions, as calculated from the exponential distribution of trajectory durations (mean 0.9 ± 0.02 s, *n* = 6314 traces). We also introduced realistic single molecule fluorescence rendering parameters for STAR635P (σ = 0.22 μm, FWHM = 0.53 μm) (Figure 1D).

### Modeling NLGN1 Diffusive and Confined Behaviors

We then performed SPT simulations of the same duration as for uPAINT experiments (2000 frames = 40 s), and analyzed the trajectories of virtual single molecules with the SPT Analysis menu in FluoSim. Using these parameters, FluoSim generated trajectory maps that mimicked experimental ones with clear confinement events inside synapses (Figure 1F), and global diffusion coefficient distributions that aligned well on the two experimental peaks (Figure 1H). Experimental distributions were somewhat more spread than theoretical ones, most likely because of local membrane heterogeneities that can contribute to NLGN1 confinement outside synapses, and/or more complex binding kinetics which are not accounted for in the model. To estimate the influence of the model parameters on the balance between mobile and confined NLGN1 populations (characterized by the relative peaks at *D*_out_ = 0.15 μm^2^/s and *D*_trap_ = 0.006 μm^2^/s, respectively), we ran a series of simulations by individually varying *D*_in_, *k*_on_, or *k*_off_, while adjusting *P*_crossing_ so as to keep a constant NLGN1 synaptic enrichment (Supplementary Figures 1, 2). The fraction of confined NLGN1 molecules increased at the expense of fast-diffusing molecules with increasing *k*_on_ or decreasing *k*_off_, i.e., either way by enhancing the trapping affinity. Changing *D*_in_ did not influence much the ratio between confined and mobile NLGN1 molecules, most likely because the fraction of freely diffusing molecules in synapses is small compared to bound ones. To evaluate the impact of changing synapse density on the histograms of NLGN1 diffusion coefficients, we kept the same dendritic geometry but varied the number of active PSDs able to trap NLGN1 (from 0 to 24), while keeping the other parameters as constant (Supplementary Figures 1, 2). This is supposed to mimic the effect of neuronal development, where the number of synapses increases with time in culture (Czöndör et al., 2012; Chanda et al., 2017). The simulations show that the fraction of confined NLGN1 molecules increases significantly with synapse density, independently of changes in binding kinetics. This result indicates that the increase in overall NLGN1 confinement observed between DIV 10 and 14 might be solely due to an increase in synapse number, and not necessarily to a change in the trapping properties of the PSD. Interestingly, those data show that although NLGN1 is enriched at synapses, a large reservoir of NLGN1 (>50%) stays mobile in the dendritic shaft.

### Transient Confinement Domains of NLGN1 at Synapses

**FIGURE 2:**
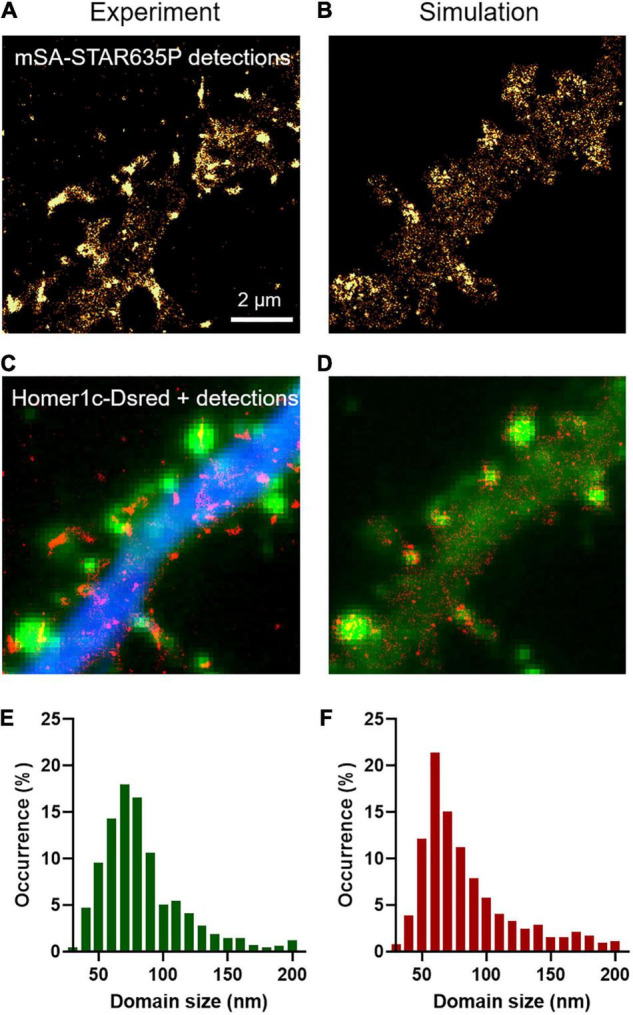
Transient confinement domains of NLGN1. **(A)** Sum of all single mSA-STAR635P localizations detected over a live sequence of 2,000 frames of 20 ms in the dendritic segment of a neuron expressing BFP + shRNA to NLGN1, AP-NLGN1, BirA^ER^, and Homer1c-DsRed. The total number of localizations was 29,303. The gold color codes for the surface density of accumulated NLGN1 molecules over time. **(B)** Corresponding simulated image of NLGN1 localizations, generated by considering the diffusional trapping of NLGN1 at PSDs and the photophysics of mSA-STAR635P emission. The total number of localizations is 29,730. **(C)** Merged image showing the integrated number of experimental single molecule localizations per pixel (red), Homer1c-DsRed (green), and BFP (blue). **(D)** Merged image showing the integrated number of simulated single molecule localizations per pixel (red) and Homer1c-DsRed (green). **(E,F)** Distribution of the size (FWHM) of the NLGN1 confinement domains obtained by experiment and simulation, respectively. The experimental and simulated distributions of domain sizes were compared by a non-parametric Mann–Whitney test (non-significant difference, *P*-value = 0.74).

In addition to providing an estimation of diffusion coefficients, uPAINT experiments can also be used to generate localization maps representing the sum of all single molecules detected over the acquisition period. For molecules that diffuse fast in the extra-synaptic space, the localization distribution is spread over a cloud of individual points, whereas for molecules that are dynamically trapped at synapses and in the dendritic shaft, the localization map forms “hot spots” that represent transient confinement domains (Figures 2A,C). The size of these domains depends on the diffusion coefficient of the molecule trapped in the synapse, the potential movement of the PSD during the acquisition period (a process called “morphing”; Blanpied et al., 2008), and the localization precision of the optical system. As a rule of thumb, the characteristic radius *r* of such domains obeys the following equation: <*r*^2^> = 4 *D*_trap_ τ_ON_, where *D*_trap_ is the diffusion coefficient of NLGN1 molecules trapped at synapses (in the order of 6 × 10^–3^ μm^2^/s) and τ_ON_ is the time during which an mSA probe emits fluorescence in uPAINT illumination conditions (t_ON_ = 1/k_OFF_^Fluo^ = 200 ms). Thus, *r* is in the range of 69 nm. As determined experimentally, the size of the NLGN1 confinement domains was 87 ± 2 nm (mean ± SEM, *n* = 688 clusters from 2 neurons) (Figure 2E), close to this theoretical estimate. To model the formation of such NLGN1 confinement domains, we uploaded FluoSim with the same set of parameters as above, and generated super-resolved maps integrating all single molecule detections throughout a live uPAINT sequence of 2,000 frames, using a zoom of 5 with respect to original images (pixel size 32 nm) and a localization precision σ = 25 nm (FWHM = 58 nm). This approach resulted in the clear visualization of confinement domains localized at the PSD where NLGN1 molecules get trapped (Figures 2B,D). The size of the domains was on average 92 ± 2 nm (*n* = 541 clusters from 12 simulations), with a statistically similar distribution as the one determined experimentally (Figure 2F).

### Long Term Turnover of NLGN1 at Synapses

**FIGURE 3:**
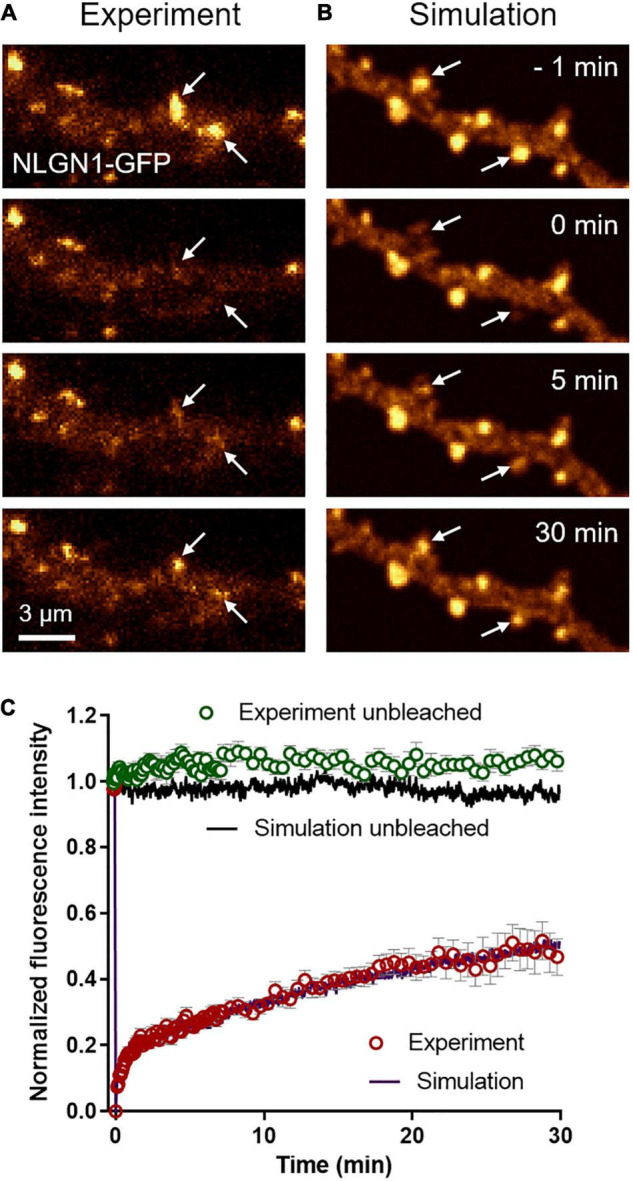
Long term turnover of NLGN1 at synapses monitored by FRAP. **(A)** Representative time sequence of a FRAP experiment performed on a neuron expressing NLGN1-GFP. The GFP signal is color coded in gold to better visualize intensity changes over time. NLGN1-GFP was photobleached at time 0 with a focused 491-nm laser beam at two specific synapses where NLGN1 was accumulated (arrows), and fluorescence recovery was monitored for 30 min. **(B)** Corresponding simulated FRAP sequence. The dendritic geometry of 135 μm^2^ was filled with 25,000 individual molecules, each with a realistic point spread function, together providing a fluorescence-like image that is also color coded in gold. The fluorescence intensity of molecules inside two PSDs was decreased by 75% at time zero (arrows), mimicking the action of the laser. **(C)** Normalized FRAP curves obtained by experiment (open circles) in bleached synapses (red, mean ± SEM of 25 synapses from 4 neurons) or unbleached synapses (green, mean ± SEM of 11 synapses from 4 neurons) and corresponding simulations (solid curves, average of 10 repetitions each, SEM < 1% mean, not shown). The Spearman correlation coefficient between experimental and simulated data for bleached synapses was 0.98, while the parameter χ^2^ estimating the goodness of fit was 0.05, both values being calculated out of 100 time points.

To characterize the long term turnover of NLGN1 at synapses, we performed FRAP experiments using a NLGN1 construct bearing an intracellular GFP tag located just below the transmembrane domain (Dresbach et al., 2004). The NLGN1-GFP protein accumulated at synapses almost as well as AP-NLGN1 labeled with mSA (Chamma et al., 2016a) (i.e., synaptic enrichment = 3.1 ± 0.2, *n* = 32 synapses from nine neurons). When photobleaching was performed on synaptic NLGN1-GFP, there was a fast initial 20% recovery that likely corresponds to diffusional exchange, followed by a slower almost linear phase that reached 50% recovery in 30 min, which reflects the continuous binding and unbinding of NLGN1 at the synapse (Figures 3A,C). Control unbleached synapses did not display any significant drop in NLGN1-GFP fluorescence, revealing negligible observational photobleaching. Additional FRAP experiments with a lower sampling rate in neurons co-expressing NLGN1-GFP and Xph20-mRuby2, an intrabody specific to PSD-95 (Rimbault et al., 2019, 2021), showed that photobleached NLGN1-GFP was essentially post-synaptic (Supplementary Figure 3A). Furthermore, the NLGN1-GFP fluorescence recovery after 1 h was 60%, a value in line with the first round of experiments performed at higher sampling rate (Supplementary Figures 3B,C).

To mimic FRAP experiments, we introduced a large number of molecules in the simulator (25,000 copies for a dendritic region of 135 μm^2^, corresponding to a surface density of 185 molecules/μm^2^) and generated fluorescence-like images by defining a Gaussian intensity profile for each GFP-tagged molecule (σ = 0.17 μm, FWHM = 0.47 μm) (Figure 3B). To induce local photo-bleaching, we chose a bleaching rate (4.0 s^–1^) reproducing the initial drop of fluorescence observed experimentally (∼75% in 500 ms). We then entered the NLGN1 extra-synaptic and synaptic diffusion coefficients (*D*_out_ and *D*_trap_) previously obtained from SPT data. We ran a series of simulations by individually varying *D*_in_, *k*_on_, or *k*_off_, while adjusting *P*_crossing_ so as to keep a constant NLGN1 synaptic enrichment of 3.5 (Supplementary Figure 4). *D*_in_ had mild effect on the simulated FRAP curve, i.e., increasing *D*_in_ slightly moved up the long term slope of the FRAP curve (Supplementary Figure 4C). Increasing *k*_on_ essentially reduced the fast recovering fraction, without changing much the long term slope (Supplementary Figure 4D). In contrast, increasing *k*_off_ dramatically accelerated the whole FRAP curve (Supplementary Figure 4E). Based on these simulated curves, we chose the best pair of coefficients that matched the experimental 30 min FRAP curve, i.e., *k*_off_ = 0.0005 s^–1^ and *k*_on_ = 0.0008 s^–1^, as well as intermediate values *D*_in_ = 0.06 μm^2^/s and *P*_crossing_ = 0.6 (Figure 3C), by minimizing a least squares function (Supplementary Figure 4F). The simulated curves also fit very well the 1 h FRAP experiment performed at lower sampling rate (Supplementary Figures 3B,C). With these parameters, the simulated images at steady state predicted NLGN1 enrichment in the post-synapse that matched experimental values (3.25 ± 0.05, *n* = 18 simulations) (unpaired *t*-test, no significant difference between experiment and simulation, *P* = 0.6). Thus, whereas uPAINT provides precise estimates of NLGN1 diffusion coefficients outside and inside synapses, FRAP experiments together with the measurement of NLGN1 synaptic enrichment allow for a determination of long-term trapping rates. Overall, the combination of single molecule and ensemble measurements offers a consistent set of parameters to model NLGN1 dynamics within the same framework.

### Nanoscale Organization of NLGN1 at Synapses

**FIGURE 4:**
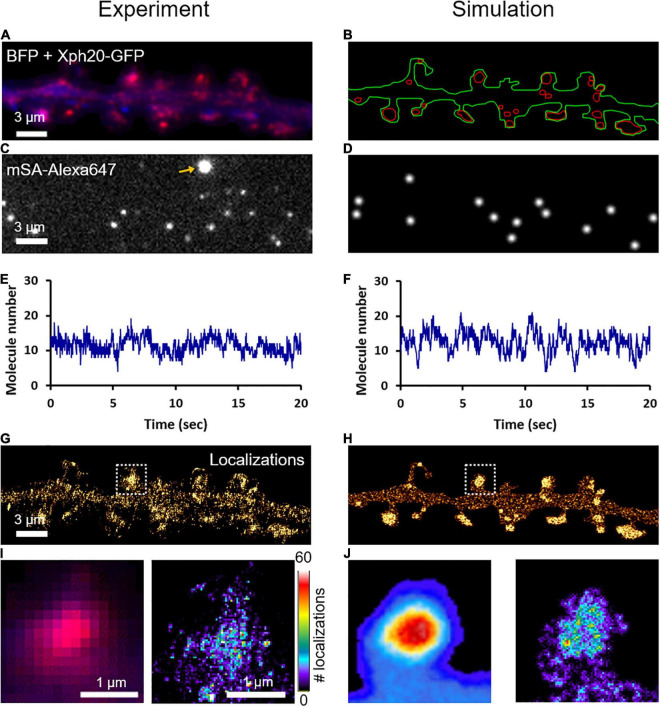
Nanoscale organization of NLGN1 at synapses characterized by dSTORM. **(A)** Dual color image of a dendritic segment from a neuron co-expressing BFP (blue) + shRNA to NLGN1, AP-NLGN1, BirA^ER^, and Xph20-GFP (red). **(B)** The BFP and Xph20-GFP images, respectively, were used as reference to draw the dendrite outline (green) and the PSDs (red areas) entered in the simulator. **(C)** Representative single frame image of a dSTORM sequence performed on AP-NLGN1 labeled with Alexa647-conjugated mSA. The yellow arrow indicates a bright fluorescent bead used to correct for drift. **(D)** Simulated image showing single molecule fluorescence emission in the same cell geometry, each with a Gaussian intensity profile. **(E,F)** Number of single molecules per frame detected in the defined geometry for experiment or simulation, respectively, and plotted over time. **(G)** Experimental super-resolved image generated from 470,821 single molecule localizations (pixel size 32 nm, total acquisition time 800 s). Note the accumulation of NLGN1 in PSDs. **(H)** Simulated super-resolved map with a localization precision of 58 nm (FWHM). The total number of single molecule detections is 498,447. **(I)** Zoom on one dendritic spine showing the low resolution merged image between BFP (blue) and PSD (magenta), and the super-resolved dSTORM image of NLGN1 distribution in false color. **(J)** Simulated heat map images showing the low and high resolution images of NLGN1 accumulation in the PSDs of the same spine. The color code for the number of single molecule localizations per pixel applies to the right images of both panels **(I,J)**.

To characterize the nanoscale organization of NLGN1 in the neuronal membrane and get access to the number of NLGN1 molecules in synapses, we performed dSTORM experiments on neurons expressing shRNA to NLGN1 plus rescue AP-NLGN1 (Figure 4A). Biotinylated AP-NLGN1 was densely labeled with Alexa647-conjugated mSA in live conditions, followed by fixation, and the stochastic emission of single fluorophores was induced (Figures 4C,E). When super-resolved images were reconstructed from individual detections, NLGN1 filled PSDs labeled with the Xph20-GFP intrabody to PSD-95 (Rimbault et al., 2019, 2021) without forming any specific sub-domain (Figures 4G,I), as previously reported (Chamma et al., 2016a). In the dendritic shaft, NLGN1 showed a fairly homogeneous membrane localization, likely corresponding to the fast-diffusing molecules detected live by uPAINT. To simulate stochastic fluorescence emission of Alexa647 dyes (Dempsey et al., 2011), we first calculated the switch-on rate (*k*_ON_^Fluo^ = 0.004 s^–1^) and switch-off rate (*k*_OFF_^Fluo^ = 6.3 s^–1^) of isolated substrate-bound mSA-Alexa647 probes in dSTORM imaging conditions (Supplementary Figure 5; Lagardère et al., 2020). To estimate the number of Alexa647 dyes conjugated per mSA, we counted the photobleaching steps of single substrate-bound mSA-Alexa647 molecules in Tyrode solution (Supplementary Figure 6). We visualized essentially one or two photobleaching steps, corresponding to an average of 1.26 Alexa dyes per mSA, in agreement with a 1.3 degree of labeling (DOL) separately measured by spectroscopy. To reproduce dSTORM experiments performed on AP-NLGN1 labeled with mSA-Alexa647, we then introduced in the imported geometry of surface area 118 μm^2^ (Figure 4B) the number of mSA molecules corresponding to the average number of experimental detections per frame (13.3) (Figures 4C–F) divided by the on-off duty cycle of mSA-Alexa647 (0.0006), giving a total of 19,843 mSA molecules (surface density = 167 molecules/μm^2^). After an equilibration period allowing NLGN1 molecules to accumulate at synapses with kinetic rates *k*_on_ = 0.0008 s^–1^ and *k*_off_ = 0.0005 s^–1^ as validated from FRAP experiments, we further set all diffusion coefficients to zero to mimic cell fixation. We then simulated the accumulation of single molecule localizations for 40,000 frames, including a realistic localization precision (σ = 25 nm, FWHM = 58 nm), to mimic the experimental super-resolved maps of NLGN1 distribution (Figures 4H,J). As expected, the overall number of single molecule detections obtained in simulations precisely matched experimental ones, thereby validating the measurement of photo-physical parameters made in parallel. In addition, simulated images faithfully reproduced the nanoscale distribution of NLGN1 outside and inside synapses observed experimentally (Figures 4G–J), and gave NLGN1 synaptic enrichment values (3.10 ± 0.08, *n* = 44 PSDs, two dendritic segments) similar to experimental ones (3.24 ± 0.12, *n* = 111 PSDs, five dendritic segments). Interestingly, we could then use the density of virtual molecules introduced in the model (19,843 mSA copies spread over the 118 μm^2^ dendritic area) as a reference to predict the average copy number of mSA molecules bound to NLGN1 at steady state in each synapse. To this aim, we just generated a single frame snapshot in the super-resolution imaging (SRI) menu of FluoSim, setting the photophysical parameters *k*_ON_^Fluo^ = 10 s^–1^ and *k*_OFF_^Fluo^ = 0 s^–1^ so as to make all fluorophores visible, then counted the number of molecules per PSD (*n* = 272 ± 20, *n* = 44 PSDs from two dendritic segments).

## Discussion

In summary, we provide here a detailed description of the surface trafficking of NLGN1 in the dendritic membrane by interpreting fluorescence live-cell and super-resolution imaging experiments using a quantitative computer software, FluoSim (Lagardère et al., 2020). The advantage of this correlative approach is that different imaging paradigms can be modeled using a small set of dynamic and photophysical parameters. Interestingly, each technique is used to feed the program with critical parameters that are not easily accessible with other imaging methods. We give a schematic overview of our strategy to estimate one by one the parameters entered in the simulator (Supplementary Figure 7). Specifically, single molecule tracking (uPAINT) provides precise mean values of NLGN1 diffusion coefficients inside and outside synapses, but because of the short duration of the trajectories, fails to capture the long-term residence time of NLGN1 in PSDs. In contrast, FRAP gives a single curve whose fit includes several unknown dynamic coefficients, but when combined with the calculation of the synaptic enrichment of NLGN1 and the diffusion coefficients inferred from uPAINT, the long range recovery provides estimates of the binding and unbinding rates of NLGN1 to the PSD scaffold. Finally, dSTORM yields static super-resolution maps of NLGN1 distribution in the dendritic membrane that can be faithfully reproduced by filling the model with a high density of molecules made immobile to mimic chemical fixation, after an equilibration period to reach steady-state distribution. Strikingly, by considering the photophysics of the Alexa647-conjugated mSA probe, the interpretation of dSTORM sequences by FluoSim provides an estimate of the molecular density of NLGN1 in the neuronal membrane, and hence of NLGN1 copy number in single PSDs.

We thereby calculate that a PSD contains on average 272 mSA molecules bound to NLGN1. Although mSA has four potential NHS conjugation sites (N-terminus plus 3 accessible lysine residues) (Chamma et al., 2017), such that each mSA molecule may carry a different number of fluorophores (from zero to 4), we made sure to use an mSA preparation in which the average number of Alexa647 dyes was close to 1 (DOL = 1.3). In any case, the DOL should not influence much the photophysical rates in dSTORM, as reported for antibodies with up to eight conjugated Alexa647 dyes (Sauer et al., 2020). Further assuming that both NLGN1 subunits are biotinylated and that each one binds an mSA probe, we estimate the presence of ∼136 NLGN1 dimers per PSD. We can moderate this number by almost a factor of two by considering the fact that, despite our NLGN1 replacement strategy, the rescue construct is most likely over-expressed by two-fold over endogenous NLGN1 (Chamma et al., 2016a; Toledo et al., 2022). In addition, according to the kinetic parameters of the model, only a 70% fraction of synaptic NLGN1 is actually bound to the PSD, while the other 30% fraction is free to diffuse in the synapse. This finally yields a value of 48 NLGN1 dimers bound to the PSD, which is 6–10 fold lower than the estimated number of PSD-95 proteins that can accommodate NLGN1 anchoring at PSDs (between 300 and 500) (Chen et al., 2005; Sheng and Hoogenraad, 2007), especially considering that other PDZ-domain containing proteins such as PSD-93, SAP-97, SAP-102, and S-SCAM can also bind NLGN1 through its C-terminal PDZ domain binding motif (Irie et al., 1997; Hirao et al., 1998). Thus, our model hypothesis that the number of PSD binding slots is in excess of NLGN1 molecules should be valid. Based on published crystal structures (Araç et al., 2007; Fabrichny et al., 2007; Chen et al., 2008), a NLGN1 dimer is likely to occupy a projected area of 5 nm × 12 nm = 60 nm^2^ in the plasma membrane, while a typical PSD has a surface area of roughly 350 nm × 350 nm, i.e., 122,000 nm^2^ (Sheng and Hoogenraad, 2007). Thus, 48 NLGN1 molecules would represent 2,880 nm^2^/122,000 nm^2^ = 2.4% of the PSD area which is a reasonable number and leaves room to many other membrane molecules including lipids, adhesion proteins, and neurotransmitter receptors (Chen et al., 2005; Lowenthal et al., 2015). In comparison, a recent proteomics study provides an estimate of 21 NLGN3 molecules per PSD (Lowenthal et al., 2015), which is in the same order of magnitude especially considering that NLGN3 are present at both excitatory and inhibitory synapses (Budreck and Scheiffele, 2007).

The rate constants *k*_on_ and *k*_off_ introduced in the model are pooled parameters that represent the overall anchorage of NLGN1 to the synapse, taking into consideration multiple protein-protein interactions, including the extracellular binding of NLGN1 to pre-synaptic NRXNs (Dean et al., 2003), and the intracellular binding to scaffolding proteins, e.g., PDZ domain containing proteins such as PSD-95 (Irie et al., 1997; Mondin et al., 2011), and potentially other non-canonical binding partners (Shipman et al., 2011). Fitting our FRAP data indicates that NLGN1 dissociates very slowly from the synapse, potentially due to the formation of parallel interactions between dimeric NLGN1 and PDZ domain containing scaffolding proteins. In any case, the calculated dissociation rate *k*_off_ is two orders of magnitude lower that the dissociation rate between purified NLGN1 and NRXN1β (Comoletti et al., 2003), indicating that extracellular NRXN-NLGN interactions are not alone responsible for NLGN1 retention at the synapse. The corresponding association rate *k*_on_ calculated by further fitting experimental NLGN1 synaptic enrichment values was also much lower than the value previously found by quantifying the detachment rate of NRXN1β-Fc coated Quantum dots from the surface of neurons over-expressing NLGN1 (Saint-Michel et al., 2009), or from FRAP experiments performed on GFP-NRXN1β accumulated at contacts with COS-7 cells expressing NLGN1-mCherry (Lagardère et al., 2020). This finding indicates that the kinetic rate *k*_on_ increases with NLGN1 expression level, as expected from a ligand-receptor reaction.

A precise evaluation of the contribution of each of these protein interactions to the actual residence time of NLGN1 at synapses will require a complete structure-function analysis of the dynamics and organization of NLGN1 mutants unable to bind specific partners. In this direction, our preliminary experiments indicate that GPI-anchored NLGN1 exhibits a diffuse localization in the dendritic membrane with no particular enrichment at post-synapses (data not shown), suggesting that the NLGN1 intracellular domain is essential for the synaptic retention of NLGN1. In addition, knocking down MDGAs as endogenous competitors of NRXN-NLGN adhesion in hippocampal neurons increases the density of excitatory synapses and reduces global NLGN1 diffusion without significantly affecting the accumulation of NLGN1 at PSDs (Toledo et al., 2022), further suggesting that the binding of NLGN1 to NRXNs does not play a major role in the synaptic retention of NLGN1. Together, these results reinforce our concept that the NLGN1 intracellular domain plays a critical role in excitatory synapse differentiation (Shipman et al., 2011; Haas et al., 2018; Letellier et al., 2018, 2020). An intracellular coupling of NLGN1 to the actin network underlying the plasma membrane (Han et al., 2017) e.g., through the WAVE regulatory complex (Chen et al., 2014), might be responsible for the transient confinement of NLGN1 in dendritic sub-domains, that was not described by our model which focuses on the selective trapping of NLGN1 at PSDs.

In addition to providing a quantitative interpretation for biological data, the simulation approach described here allows a better understanding of some subtle experimental findings linked to SRI. For example, the projection of all individual molecule detections obtained in uPAINT provides a super-resolved image where hot spots of protein localization appear, corresponding to the confinement domains of a subset of molecules. Because of the live imaging conditions and the intrinsic movement of synapses, the localization of these domains can evolve over time (Nair et al., 2013). These objects are thus different from the static protein “nanodomains” that can be identified from stimulated emission depletion (STED) or dSTORM images acquired after saturating protein labeling and chemical fixation (Nair et al., 2013; Chamma et al., 2016a,b; Tang et al., 2016; Hruska et al., 2018). Things can be complicated even more by the existence of synapses containing multiple PSDs (Hruska et al., 2018). Thus, care must be taken in interpreting super-resolution images, and computer simulations can be helpful to put realistic values on the numbers of labeled molecules, the photophysical parameters behind single molecule fluorescence emission peaks, and the time frame of the acquisition sequences, that are all susceptible to affect the actual representation of the imaging data. Finally, one limitation of FluoSim is that it is currently constrained to the simulation of 2D images, while the actual dynamics of membrane molecules including NLGN1 takes place in more complex 3D geometries such as the surface of a dendrite. Theoretical analyses have been published that estimate the error made by approximating 3D diffusion by a 2D diffusion coefficient (Renner et al., 2011). This type of correction might be applied while waiting for a 3D version of the simulator in combination with 3D single molecule tracking of NLGN1.

## Materials and Methods

### DNA Plasmids and Proteins

shRNA to NLGN1 (Chih et al., 2005) containing a GFP reporter was a gift from P. Scheiffele (Biozentrum, Basel, Switzerland). shRNA to NLGN1 containing a BFP reporter was described earlier (Toledo et al., 2022). NLGN1 with GFP insertion at position 728 below the transmembrane domain (Dresbach et al., 2004) was a gift from T. Dresbach (University Medical Center, Göttingen, Germany). AP-NLGN1 and BirA^ER^ (Howarth et al., 2005) were gifts from A. Ting (Stanford University, Palo Alto, CA, United States). Homer1c-DsRed was described earlier (Mondin et al., 2011). shRNA-resistant AP-NLGN1 was described earlier (Chamma et al., 2016a; Toledo et al., 2022). The specific intrabody to PSD-95, Xph20 (Addgene ID 135530) was described recently (Rimbault et al., 2019, 2021), and we used both GFP- and mRuby-tagged versions. The bacterial production of mSA, purification, and conjugation to organic dyes (STAR635P or Alexa647) to a final DOL comprised between 0.6 and 2 (dye to protein ratio), were described previously (Chamma et al., 2017).

### Rat Hippocampal Cultures and Electroporation

Gestant Sprague-Dawley rat females were purchased from Janvier Labs (Saint-Berthevin, France). Animals were handled and killed according to European ethical rules. Dissociated neuronal cultures were prepared from E18 rat embryos as previously described (Kaech and Banker, 2006). Dissociated cells were electroporated with the Amaxa system (Lonza, Basel, Switzerland) using 300,000 cells per cuvette. The following plasmid combinations were used. For uPAINT: Homer1c-DsRed: shNLGN1-GFP: AP-NLGN1rescue: BirA^ER^ (1:1:1:1 μg DNA). For dSTORM: Xph20-GFP: shNLGN1-EBFP: AP-NLGN1rescue: BirA^ER^ (1:1:1:1 μg DNA). For FRAP, NLGN1-GFP (3 μg DNA) or NLGN1-GFP: Xph20-mRuby2 (1:1 μg DNA). Electroporated neurons were resuspended in Minimal Essential Medium (Thermo Fisher Scientific, Illkirch, France #21090.022) supplemented with 10% Horse serum (Invitrogen, Illkirch, France) (MEM-HS), and plated on 18 mm glass coverslips coated with 1 mg/mL polylysine (Sigma-Aldrich, Saint-Quentin-Fallavier, France #P2636) overnight at 37°C. Three hours after plating, coverslips were flipped onto 60 mm dishes containing 15 DIV rat hippocampal glial cells cultured in Neurobasal plus medium (Gibco, Illkirch, France, #A3582901) supplemented with 2 mM glutamine and 1x B27™ plus Neuronal supplement (Gibco, Illkirch, France, #A3582801). Neurons were cultured during 10–14 days at 37°C and 5% CO_2_. Astrocyte feeder layers were prepared from the same embryos, plated between 20,000 and 40,000 cells per 60 mm dish previously coated with 0.1 mg/mL polylysine and cultured for 14 days in MEM containing 4.5 g/L glucose, 2 mM L-glutamax (Sigma-Aldrich, Saint-Quentin-Fallavier, France #3550-038) and 10% horse serum. Ara C (Sigma-Aldrich, Saint-Quentin-Fallavier, France #C1768) was added after 3 DIV at a final concentration of 3.4 μM.

### Single Molecule Tracking (uPAINT Experiments)

Universal point accumulation in nanoscale topography (uPAINT) was carried out as reported (Giannone et al., 2010; Chamma et al., 2016a). Neurons at DIV 10 or 14 were mounted in Tyrode solution (15 mM D-glucose, 108 mM NaCl, 5 mM KCl, 2 mM MgCl_2_, 2 mM CaCl_2_ and 25 mM HEPES, pH 7.4) containing 1% globulin-free BSA (Sigma-Aldrich, Saint-Quentin-Fallavier, France, #A7638) in an open Inox observation chamber (Life Imaging Services, Basel, Switzerland). The chamber was placed on a motorized inverted microscope (Nikon Ti-E Eclipse) equipped with perfect focus system and an APO TIRF 100x/1.49 NA oil immersion objective, and enclosed in a thermostatic box (Life Imaging Services, Basel, Switzerland) providing air at 37°C. Neurons co-expressing shRNA to NLGN1 containing a GFP reporter and Homer1c-DsRed were detected using a mercury lamp (Nikon Xcite) and the following filter sets (Semrock, Rochester, NY, United States): EGFP (Excitation: FF01-472/30; Dichroic: FF-495Di02; Emission: FF01-525/30) and DsRed (Excitation: FF01-543/22; Dichroic: FF-562Di02; Emission: FF01-593/40). Recombinant AP-NLGN1 biotinylated by BirA^ER^ was sparsely labeled using a low concentration of STAR635P-conjugated mSA (1 nM). A four-color laser bench (405/488/561 nm lines, 100 mW each; Roper Scientific, Evry, France and 1 W 647 nm line, MPB Communications Inc., Pointe-Claire, QC, Canada) is connected through an optical fiber to the Total Internal Reflection Fluorescence (TIRF) illumination arm of the microscope. Laser power was controlled through an acousto-optical tunable filter (AOTF) driven by the Metamorph software (Molecular Devices, San Jose, CA, United States). STAR635P was excited with the 647 nm laser line (∼2 mW at the objective front lens), through a four-band beam splitter (BS R405/488/561/635, Semrock, Rochester, NY, United States). Samples were imaged by oblique laser illumination, allowing the excitation of individual mSA-STAR635P molecules bound to the cell surface, without illuminating probes in solution. Fluorescence was collected on an EMCCD camera with 16 μm pixel size (Evolve, Roper Scientific, Evry, France), using a FF01-676/29 nm emission filter (Semrock, Rochester, NY, United States). Stacks of 2,000 consecutive frames were obtained from each cell with an integration time of 20 ms. Images were analyzed using PALM-Tracer, a program running on Metamorph and based on wavelet segmentation for molecule localization and simulated annealing algorithms for tracking (generously provided by J. B. Sibarita, Bordeaux) (Izeddin et al., 2012). This program allows for the tracking of localized molecules through successive images. Trajectories longer than 10 frames (200 ms) were selected. The diffusion coefficient, D, was calculated for each trajectory, from linear fits of the first 4 points of the mean square displacement (MSD) function versus time. Trajectories with displacement inferior to the pointing accuracy (∼50 nm in uPAINT conditions) whose MSD function cannot be properly fitted are arbitrarily placed at *D* = 10^–5^ μm^2^ s^–1^.

### Direct Stochastic Optical Reconstruction Microscopy Experiments

Neurons co-expressing shRNA to NLGN1 containing an EBFP reporter, Xph20-GFP, rescue AP-NLGN1, and BirA^ER^ were surface-labeled with a high concentration (100 nM) of Alexa647-conjugated mSA in Tyrode solution containing 1% globulin-free BSA (Sigma-Aldrich, Saint-Quentin-Fallavier, France, #A7638) for 10 min, rinsed and fixed with 4% PFA-0.2% glutaraldehyde in PBS for 10 min at room temperature, and stored in PBS at 4°C until imaging (within a few days). Cells were imaged in Tris-HCl buffer (pH 7.5), containing 10% glycerol, 10% glucose, 0.5 mg/mL glucose oxidase (Sigma-Aldrich, Saint-Quentin-Fallavier, France, #G2133), 40 mg/mL catalase (Sigma-Aldrich, Saint-Quentin-Fallavier, France, #C100-0,1% w/v) and 50 mM β-mercaptoethylamine (MEA) (Sigma-Aldrich, Saint-Quentin-Fallavier, France, #M6500) (Heilemann et al., 2008). The same microscope described for uPAINT was used. Detection of the EBFP reporter was made with the following filter set from Semrock, Rochester, NY, United States (Excitation: FF02-379/34; Dichroic: FF-409Di03; Emission: FF01-440/40). Pumping of Alexa647 dyes into their triplet state was performed for several seconds using ∼60 mW of the 647 nm laser at the objective front lens. Then, a lower power (∼20 mW) was applied to detect the stochastic emission of single-molecule fluorescence, which was collected using the same optics and detector as described above for uPAINT. 10 streams of 4,000 frames each were acquired at 50 Hz. Multi-color 100-nm fluorescent beads (Tetraspeck, Invitrogen, Illkirch, France) were used to register long-term acquisitions and correct for lateral drift. The localization precision of our imaging system in dSTORM conditions is around 60 nm (FWHM). Stacks were analyzed using the PALM-Tracer program, allowing for the reconstruction of a unique super-resolved image of 32 nm pixel size (zoom 5 compared to the original images) by summing the intensities of all single molecules localized (1 detection per frame is coded by an intensity value of 1).

### Fluorescence Recovery After Photobleaching Experiments and Analysis

Neurons expressing NLGN1-GFP were mounted in Tyrode solution, and observed under the same set-up used for uPAINT and dSTORM. The laser bench has a second optical fiber output connected to an illumination device containing two *x/y* galvanometric scanning mirrors (ILAS, Roper Scientific, Evry, France) steered by MetaMorph. It allows precise spatial and temporal control of the focused laser beam at any user-selected region of interest (ROI) within the sample for targeted photo-bleaching. Switching between the two fibers for alternating between imaging and bleaching is performed in the ms time range using an AOTF. Oblique illumination was performed using the 491 nm beam at low power (0.3 mW at the front of the objective) to image NLGN1-GFP molecules in the plasma membrane close to the substrate plane. After acquiring a 10 sec baseline at 0.5 Hz frame rate, rapid selective photo-bleaching of several synapses was achieved by scanning circular ROIs of diameter 2 μm at higher laser power (3 mW at the objective front lens), during 500 ms. Fluorescence recovery was then recorded immediately after the bleach sequence for 30 min. The recording period included three phases with decreasing frame rate ranging from 2 to 0.1 Hz. Observational photo-bleaching was kept very low, as assessed by observing control unbleached areas nearby. FRAP curves were obtained by computing the average intensity in the photobleached area, after background subtraction, and normalized between 1 (baseline) and 0 (time zero after photo-bleaching). In some experiments performed at lower sampling rate, several synapses from neurons expressing NLGN1-GFP + Xph20-mRuby2 were photobleached at time zero, and fluorescence recovery was monitored every 15 min, up to 1 hr.

### Description of FluoSim Algorithm and Parameters

A thorough description of the FluoSim algorithm together with a detailed user manual have been previously published (Lagardère et al., 2020). We give below a general outline of the software and the important parameters used in each simulation mode (SPT, STORM, and FRAP). The contour of a 48 μm-long dendritic segment containing 23 PSDs was drawn in Metamorph using an image of a 14 DIV neuron expressing Homer1c-DsRed, and saved as a region file. This region was imported in FluoSim and randomly populated with NLGN1 molecules (1,500–25,000 copies depending on the experiment to model). Those molecules are kept within the dendrite boundaries by introducing rebound conditions. An individual molecule is characterized by its 2D coordinates x and y over time t, and its intensity. The time step Δ*t* and total duration of the simulations T is set according to the experiment to model (SPT: Δ*t* = 20 ms, *T* = 40 s; dSTORM: Δ*t* = 20 ms, T = 800 s; and FRAP: Δ*t* = 100 ms, *T* = 30–60 min). The initial position of a freely diffusing molecule is defined by *x*(0) = *x*_0_ and *y*(0) = *y*_0_, taken as random numbers to fall within the dendrite boundaries. The diffusion coefficient outside synapses (*D*_*out*_) is chosen around 0.15 μm^2^/s, based on SPT data, while synapses are characterized by a lower diffusion coefficient (*D*_*in*_ = 0.06 μm^2^/s), owing to molecular crowding. An additional coefficient called crossing probability describes the potentially limited penetrability of molecules into the synapse because of steric hindrance (*P*_*crossing*_ = 0.5). A 20% fraction of immobile NLGN1 molecules was observed in uPAINT (with *D* = 10^–5^ μm^2^/s) and introduced in the program at random positions with zero diffusion coefficient. In the synapse, NLGN1 molecules are allowed to bind reversibly to the quasi-static PSD scaffold, with first order binding and unbinding rates *k*_*on*_ and *k*_*off*_, respectively (both in s^–1^). The *k*_*on*_ and *k*_*off*_ values were obtained by fitting FRAP experiments. NLGN1 bound to the PSD was allowed to diffuse at a lower diffusion coefficient *D_*trap*_* = 0.006 μm^2^/s, reflecting slow PSD morphing over time (Blanpied et al., 2008). The number of PSD binding sites is assumed to be in excess, such that the binding rate *k*_*on*_ is maintained constant throughout the simulations, i.e., it does not depend on the number of NLGN1 molecules recruited at synapses over time. We further consider a non-discrete distribution of binding sites in the PSD, consistent with our previous observation that NLGN1 does not tend to form nanodomains and fills the PSD rather uniformly (Chamma et al., 2016a).

#### Calculation of Positions

At each time step, the (*x,y*) coordinates of each molecule are incremented by the distances (Δ*x*, Δ*y*), which depend on whether the molecule is outside or inside the synapse, or bound to the PSD. If the molecule is extra-synaptic, it follows a random walk with diffusion coefficient *D*_*out*_. The positions *x*(t) and *y*(t) are then incremented at each time step by *n*_ x_(2*D*_*out*_Δ*t*)^1/2^ and *n*_ y_(2*D*_*out*_Δ*t*)^1/2^, respectively, where *n*_ x_ and *n*_ y_ are random numbers generated from a normal distribution with zero mean and variance unity, to account for the stochastic nature of diffusion. This ensures that the mean square displacement stays proportional to time, i.e., <*x*^2^ +*y*^2^> = 4*D_*out*_t*. If the adhesion molecule reaches a synapse, it is set to diffuse with a lower diffusion coefficient *D*_*in*_, with increments n_x_(2*D*_*in*_Δ*t*)^1/2^ and n_y_(2*D*_*in*_Δ*t*)^1/2^. Whenever the molecule resides in the synapse, it is allowed to bind to the PSD only if the probability of coupling in this time interval, *P_*coupl*_* = *k*_*on*_Δ*t*, is greater than a random number *N* between 0 and 1 generated from a uniform distribution. If this is not the case, the molecule continues to diffuse until both conditions are met, i.e., the molecule remains in the synapse and the probability of binding is greater than the random number *N*, differently chosen at each time increment. Upon binding, NLGN1 is set to diffuse with a slow diffusion coefficient *D*_*trap*_, thus the positions *x*(t) and y(t) are incremented by *n*_ x_(2*D*_*trap*_Δ*t*)^1/2^ and n_y_(2*D*_*trap*_Δ*t*)^1/2^, respectively. NLGN1 stays bound until the probability for dissociation *P_*detach*_* = *k*_*off*_Δ*t*, exceeds another random number *N′*. It then binds again or escapes into the extra-synaptic space. An option is proposed in FluoSim to theoretically estimate the steady-state, by placing more molecules in synapses, considering both slower diffusion and adhesion. The theoretical NLGN1 synaptic enrichment is then given by the formula (*P_*crossing*_ × D_*out*/_D_*in*_*) (1 + *k*_*on*_/*k*_*off*_).

#### Molecule Size, Intensity, and Photophysics

In addition to its position, each molecule is defined by its size and fluorescence intensity over time. Single molecules are represented either by a discrete point of intensity 1, or by a Gaussian intensity profile with a peak value directly coded on a 16-bit gray scale (0-65535 levels), or expressed in photons/sec associated with a conversion rate, or gain, which gives the number of gray levels read on the virtual camera chip per incoming photon. The Gaussian representation comprises an adjustable width σ (the standard deviation) in the order of λ/(2 × N.A.), where λ is the emission wavelength of the fluorophore, and N.A. is the numerical aperture of the objective (1.49 in our set-up). The corresponding FWHM is then equal to 2σ√(2.ln2) (Deschout et al., 2014). In our experiments, we used NLGN1-GFP: σ_GFP_ = 510/(2 × 1.49) = 171 nm, and FWHM_GFP_ = 402 nm, STAR635P-conjugated mSA: σ_STAR635P_ = 651/(2 × 1.49) = 218 nm and FWHM_STAR635P_ = 529 nm, and Alexa647-conjugated mSA: σ_A647_ = 668/(2 × 1.49) = 224 nm and FWHM_A647_ = 527 nm. Transitions between ON/OFF intensity values are set by two photo-physical parameters: the switch-on rate (*k_*ON*_*^Fluo^**) and the switch-off rate (*k_*OFF*_*^Fluo^**). These rates are in units of sec^–1^ and represent the probabilities per unit of time that a molecule switches from a state where it emits fluorescence, to a state where it does not emit fluorescence, and vice versa. In uPAINT, *k_*ON*_*^Fluo^** represents the rate of binding of fluorescent mSA ligand in solution to NLGN1 molecules on the cell surface, which spontaneously appear in the oblique illumination plane, whereas *k_*OFF*_*^Fluo^** combines fluorophore photo-bleaching and probe detachment from the cell surface. To mimic a FRAP experiment, *k_*OFF*_*^Fluo^** is set to a high level in a given ROI for a few frames (500 ms) to quickly and irreversibly photo-bleach fluorophores, then recovery is monitored. In dSTORM, k_*ON*_^*Fluo*^ represents the frequency of stochastic fluorescence emission, and k_*OFF*_*^Fluo^* the inverse of the lifetime of the fluorescence emission peaks.

##### Single Particle Tracking Simulations

To mimic the sparse density of NLGN1 bound to mSA-STAR635P as used in uPAINT experiments, a relatively low number of molecules were introduced in the model cell (1,500 molecules per dendrite area of 36 μm^2^, corresponding to a surface density of 42 molecules/μm^2^). The off-rate of the simulated trajectories was adjusted by fitting the experimental distribution of trajectory durations with an exponentially decreasing function, giving *k_*OFF*_*^Fluo^** = 5.4 s^–1^ (mean trajectory duration = 220 ms). The parameter *k_*ON*_*^Fluo^** which determines the number of fluorescent molecules per frame was set to 0.03 s^–1^, so as to yield approximately the same density of visible molecules per surface area as in the experiments (0.25 molecule/μm^2^). Sequences of 2,000 frames were generated as in the experiments, and only trajectories longer than 10 frames were selected. Trajectories containing the spatial positions and intensity of each molecule over time are saved as .trc files, and can be loaded later for offline visualization and analysis (menu SPT Analysis). The diffusion coefficient, *D*, was calculated for each trajectory, from linear fits of the first four points of the MSD function versus time. Five independent simulations were run for each set of parameters, allowing the construction of histograms of diffusion coefficients directly comparable to SPT experiments.

##### Fluorescence Recovery After Photobleaching Simulations

To match the dense distribution of NLGN1 molecules that characterize FRAP experiments, a relatively large number of molecules was introduced in the virtual cell (25,000 molecules in a dendritic segment of 135 μm^2^, corresponding to a surface density of ∼148 molecules/μm^2^). Simulations of 18,200 frames, including a baseline of 200 frames, were generated with a time step of 100 ms (total duration 30 min) and a sampling rate of 2 s. The photo-activation rate was set to a maximal value (*k_*ON*_*^Fluo^** = 10 s^–1^), i.e., all molecules are initially fluorescent, while the photo-bleaching rate is set to zero during baseline and recovery acquisition (i.e., observational photo-bleaching is neglected). During the short photo-bleaching period (500 ms) applied to four PSDs, the photo-bleaching rate was set to *k_*off*_*^Bleach^** = 4.0 s^–1^ for five frames, to precisely match the initial drop of fluorescence observed experimentally (∼75%). The number of molecules in the photo-bleached PSDs and in four control unbleached PSDs was computed over time, saved as a.txt file, and normalized between 1 (baseline number of fluorescent molecules before photo-bleaching) and 0 (number of fluorescent molecules right after photo-bleaching). FRAP simulations were repeated 10 times, and the corresponding curves were averaged. To estimate the goodness of fit between simulated and experimental FRAP curves, we calculated the coefficient χ^2^ = (1/n) Σ_i_ [(F_i_^exp^ – F_i_^sim^)/σ_i_]^2^, where n is the number of experimental values, *i* = 1 to n is the time point, F_i_ are the normalized fluorescence intensity values for both experiment (exp) and simulations (sim), and σ_i_ is the standard deviation of the experimental value.

##### Direct STochastic Optical Reconstruction Microscopy Simulations

The switch-on rate *k_*ON*_*^Fluo^** at which fluorescent dyes spontaneously emit light was determined by measuring the fluorescence intensity collected from single Alexa647-conjugated mSA molecules bound to the glass coverslip during a dSTORM sequence, and counting the number of peaks (mean ± SEM = 1.7 ± 0.2 peaks over a time period of 400 s, *n* = 43 molecules analyzed, giving *k_*ON*_*^Fluo^** = 0.004 s^–1^). The switch-off rate *k_*OFF*_*^Fluo^** was determined by fitting the distribution of the time durations during which single Alexa647-conjugated mSA emitted light before entering again the non-emitting state with an exponentially decreasing function (average 11.1 ± 1.4 frames of 20 ms, 72 events analyzed), giving a value *k_*OFF*_*^Fluo^** = 6.3 s^–1^. The on-off duty cycle δ = *k_*ON*_*^Fluo^**/(*k_*ON*_*^Fluo^** + *k_*OFF*_*^Fluo^**) is the fraction of time that fluorophores spend in the light-emitting state, and equals here 0.00067, very close to reported values for isolated Alexa647-conjugated anti-GFP nanobody (Lagardère et al., 2020) and for single Alexa647 dyes in MEA-based dSTORM buffer (Dempsey et al., 2011), thereby confirming that the fluorophore to protein ratio of our conjugates is around 1. The average number of experimentally detected mSA-Alexa647 molecules per plane in the neuronal contour was *N* = 13.3, corresponding to a total number N/δ = 19,843 actual molecules in the cell geometry that was imaged (118 μm^2^), thus representing a density of 167 molecules/μm^2^. To mimic dSTORM experiments that rely on the saturating labeling of biotinylated AP-NLGN1 with mSA-Alexa647, we thus introduced 19,843 molecules in the virtual dendritic segment. After the diffusion/trapping steady-state has been imposed, the simulation was paused and all diffusion coefficients were set to zero to mimic cell fixation. Then, simulations were run for 40,000 frames of 20 ms each (total time of 800 s), and a single 16-bit image was generated which contained the integration of all molecule localizations throughout time. Three parameters are used to render the super resolution image: the intensity associated with a single detection; the zoom factor which is the ratio between the pixel sizes of the super-resolved image and the low resolution reference picture (a fivefold zoom corresponds to a pixel size of 32 nm in the high resolution image); and the localization precision, which corresponds to the standard deviation of the Gaussian distribution used to spread detections around the theoretical position of the molecule (σ = 25 nm, FWHM = 58 nm). A single super-resolved image integrating all single molecule localizations is exported as a TIFF file. To estimate mSA copy numbers in PSDs at steady-state, a single TIFF image was generated from the SRI menu of FluoSim, after setting the coefficients *k_*ON*_*^Fluo^** = 10 s^–1^ and *k_*OFF*_*^Fluo^** = 0 s^–1^ so as to visualize all emitting fluorophores. The image was then opened in Metamorph and intensity values were read in PSDs defined by previously saved ROIs.

## Data Availability Statement

The original contributions presented in the study are included in the article/Supplementary Material, further inquiries can be directed to the corresponding author.

## Ethics Statement

The animal study was reviewed and approved by the authors declare that they have complied with all relevant ethical regulations (study protocol approved by the Ethical Committee of Bordeaux CE50).

## Author Contributions

ML developed FluoSim. AD performed dSTORM experiments, long-term FRAP experiments and simulations. MS provided reagents and scientific insight. OT supervised the work, performed simulations, and wrote the manuscript. All authors reviewed the manuscript.

## Conflict of Interest

The authors declare that the research was conducted in the absence of any commercial or financial relationships that could be construed as a potential conflict of interest.

## Publisher’s Note

All claims expressed in this article are solely those of the authors and do not necessarily represent those of their affiliated organizations, or those of the publisher, the editors and the reviewers. Any product that may be evaluated in this article, or claim that may be made by its manufacturer, is not guaranteed or endorsed by the publisher.

## Supplementary Material

Supplementary Figure 1 Impact of kinetic parameters and synapse density on global NLGN1 diffusion as predicted by FluoSim. **(A)** Dendritic geometry used in FluoSim, based on the image of a neuron expressing Homer1c-DsRed (in white). The green contour is the outline of the dendrite (135 μm^2^), the red PSDs are considered active (i.e., able to trap NLGN1), while the blue PSDs are considered inactive (i.e., unable to trap NLGN1). **(B)** Image of all NLGN1 trajectories simulated in 2000 frames, with a color code representing the diffusion coefficient (red = fast diffusion, yellow = slow diffusion). Note the slower NLGN1 diffusion in active PSDs. **(C–F)** Semi-log plots showing the distribution of NLGN1 diffusion coefficients obtained by simulation, for individual variations of the parameters *D*_in_, *k*_on_, and *k*_off_, respectively (all other parameters being kept constant), or for different values of synapse density, i.e., obtained by varying the number of active PSDs. Curves were plotted from 985 to 1082 simulated trajectories per condition, obtained for 2500 NLGN1 molecules introduced in the geometry. The NLGN1 confined fraction is defined as the gray zone between –4 < Log(D) < 1.8.

Supplementary Figure 2 Impact of kinetic parameters and synapse density on the simulated NLGN1 confined fraction. **(A–D)** Plots showing the fraction of confined NLGN1 molecules as a function of the parameters *D*_in_, *k*_on_, and *k*_off_, or synapse density. Values correspond to the population highlighted in gray in Supplementary Figure 1. When not varied, parameters were *D*_out_ = 0.15 μm^2^.s^–1^, *D*_in_ = 0.15 μm^2^.s^–1^, *D*_trap_ = 0.006 μm^2^.s^–1^, *k*_on_ = 0.0015 s^–1^, *k*_off_ = 0.0005 s^–1^, and synapse density = 0.4 μm^–2^ (i.e., all PSDs were active). *P*_crossing_ was adjusted so as to maintain a constant theoretical synaptic enrichment (*P**_*crossing*_ D_*out/*_D_*in*_*) (1 + *k*_*on*_/*k*_*off*_) = 4.

Supplementary Figure 3 Long term FRAP on NLGN1-GFP. **(A)** Post-synaptic distribution of NLGN1-GFP. Representative images of a dendritic segment from a neuron co-expressing NLGN1-GFP (green) and Xph20-mRuby2 (red), an intrabody to PSD-95. The merged image shows extensive colocalization between the two proteins at post-synapses (yellow signal). **(B)** Representative 1-hr FRAP sequence performed on a neuron co-expressing NLGN1-GFP and Xph20-mRuby2. The NLGN1-GFP signal was photobleached at time 0 on two post-synapses, and epifluorescence images were acquired after 30 and 60 min. **(C)** Graph showing the fluorescence intensity of unbleached and photobleached synapses, normalized to the baseline level before photobleaching. Data are mean ± SD for 47 and 54 bleached and unbleached synapses, respectively, out of 9 neurons from 2 different experiments. The curves are computer simulations run for 1 h. The parameter χ^2^ estimating the goodness of fit calculated for the two experimental points (30 and 60 min) is 0.31 for bleached synapses and 0.014 for unbleached synapses.

Supplementary Figure 4 Impact of kinetic parameters on simulated FRAP curves. **(A)** Dendritic geometry used in FluoSim, based on the image of a neuron expressing Homer1c-DsRed. PSDs outlined in red are those which are photobleached at time zero, while PSDs outlined in blue serve as unbleached controls. **(B)** Heat map of NLGN1 localization with a color code representing the accumulation of NLGN1 in PSDs. **(C–E)** FRAP curves obtained for individual variations of the parameters *D*_in_, *k*_on_, and *k*_off_, respectively, all other parameters in FluoSim being kept constant. When not varied, parameters were *D*_out_ = 0.15 μm^2^.s^–1^, *D*_in_ = 0.15 μm^2^.s^–1^, *D*_trap_ = 0.006 μm^2^.s^–1^, *k*_on_ = 0.0015 s^–1^, *k*_off_ = 0.0005 s^–1^, and synapse density = 0.4 μm^–2^ (i.e., all PSDs were active). *P*_crossing_ was adjusted so as to maintain a constant theoretical synaptic enrichment (*P**_*crossing*_ D_*out/*_D_*in*_*) (1 + *k*_*on*_/*k*_*off*_) = 4. **(F)** Graph showing the coefficient χ^2^ estimating the goodness of fit, plotted as a function of *k*_off_. The minimum of this curve indicates the k_*off*_ value giving the best fit, which was chosen thereafter in the model.

Supplementary Figure 5 Photophysical properties of Alexa647-conjugated mSA in dSTORM conditions. **(A)** Maximum intensity projection image of a dSTORM sequence run on diluted mSA-Alexa647 molecules (1 nM) immobilized on a glass coverslip. The region of interest is 64 × 64 pixels (10 × 10 μm) and the sequence was 20,000 frames at a 20 ms exposure time per frame (total duration 400 s). **(B)** Representative single frame images of individual molecules while they emit fluorescence. **(C)** Fluorescence intensity in gray levels over time for three arbitrary regions of 6 × 6 pixels centered on individual molecules. One or several emission peaks can be clearly distinguished. The frequency of fluorescence emission by individual mSA-Alexa647 molecules, *k*_ON_^Fluo^, was taken as the average number of peaks divided by the total sequence duration (*k*_ON_^Fluo^ = 0.004 s^–1^). **(D)** Zoom on one of the peaks (molecule 1, green), showing the lifetime of the fluorescence emission. **(E)** Distribution of the fluorescence lifetime calculated from 73 individual emission peaks (black circles). The red curve is a fit with the exponentially decreasing function N_0_ Exp(–*k*_OFF_^Fluo^*t*), where N_0_ is the number of values at time zero, *t* is the time in sec, and k_OFF_^Fluo^ = 6.3 s^–1^ is the characteristic rate constant.

Supplementary Figure 6 Estimation of the number of Alexa647 fluorophores per mSA protein by quantification of photobleaching steps. Alexa-647 conjugated mSA molecules were diluted to 3 nM in Tyrode solution, immobilized on a glass substrate, and imaged by TIRF microscopy at 30 Hz. **(A)** Representative time lapse images. Note the progressive photobleaching of the mSA molecules. **(B–D)** Representative fluorescence intensity profiles of individual molecules over time. Each color represents a different molecule. Molecules display mostly 1-step **(B)** and 2-step **(C)**, but very rarely 3-step **(D)** photobleaching profiles. **(E)** Distribution of the number of photobleaching steps computed from 250 individual Alexa647-conjugated mSA molecules. The red curve represents a binomial distribution with 4 binding sites and conjugation probability of Alexa647 to mSA of 0.35. The best fit allows the computation of a 17% fraction of unconjugated mSA, thereby undetectable by fluorescence microscopy.

Supplementary Figure 7 General strategy to estimate model parameters entered in FluoSim. **(A)** Single molecule tracking experiments yield with relatively high precision the NLGN1 immobile fraction, the peak NLGN1 diffusion coefficient outside synapses (*D*_out_), and the peak diffusion coefficient of NLGN1 molecules bound at PSDs (*D*_trap_). **(B)** The fit of FRAP experiments with the model allows for the determination of the binding and unbinding rates (*k*_on_ and *k*_off_, respectively) of NLGN1 to the PSD. Further adjustment of the remaining parameters P_*c*_ and D_*in*_ are based on the determination of NLGN1 synaptic enrichment. **(C)** Quantification of mSA-Alexa647 photophysical rates in dSTORM conditions combined with the other parameters of the model allows for the determination of the copy number of NLGN1 in the dendrite geometry, by matching the absolute number of single molecule detections obtained experimentally.
