# Supplementary figures and images for: High-Resolution Fluorescence Imaging Combined With Computer Simulations to Quantitate Surface Dynamics and Nanoscale Organization of Neuroligin-1 at Synapses
Source: Front Synaptic Neurosci. 2022 Apr 25;14:835427. doi: 10.3389/fnsyn.2022.835427 (PMC9083120; doi:10.3389/fnsyn.2022.835427)

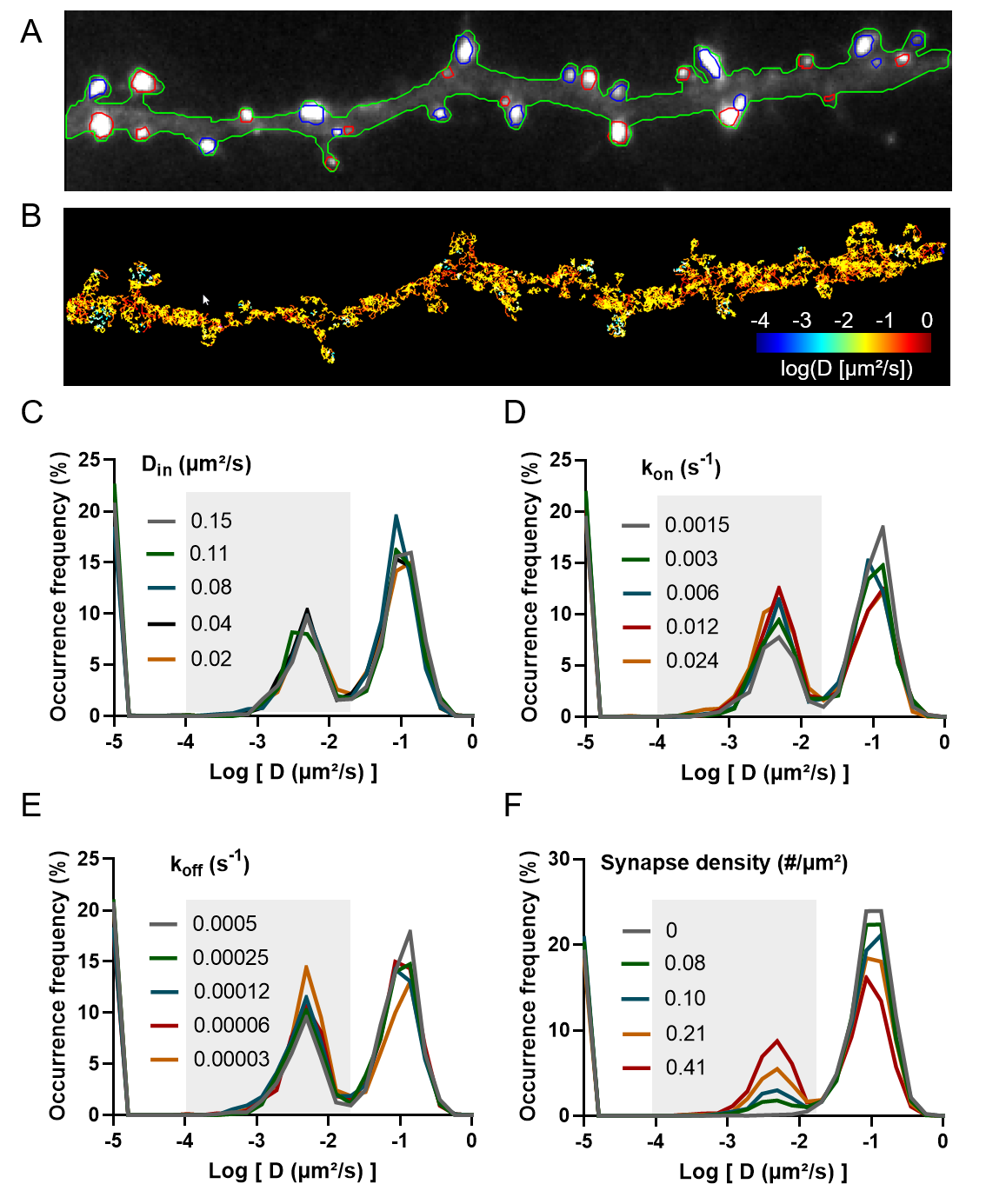

Supplement: Supplementary Figure 1 — Impact of kinetic parameters and synapse density on global NLGN1 diffusion as predicted by FluoSim. (A) Dendritic geometry used in FluoSim, based on the image of a neuron expressing Homer1c-DsRed (in white). The green contour is the outline of the dendrite (135 μm2), the red PSDs are considered active (i.e., able to trap NLGN1), while the blue PSDs are considered inactive (i.e., unable to trap NLGN1). (B) Image of all NLGN1 trajectories simulated in 2000 frames, with a color code representing the diffusion coefficient (red = fast diffusion, yellow = slow diffusion). Note the slower NLGN1 diffusion in active PSDs. (C–F) Semi-log plots showing the distribution of NLGN1 diffusion coefficients obtained by simulation, for individual variations of the parameters Din, kon, and koff, respectively (all other parameters being kept constant), or for different values of synapse density, i.e., obtained by varying the number of active PSDs. Curves were plotted from 985 to 1082 simulated trajectories per condition, obtained for 2500 NLGN1 molecules introduced in the geometry. The NLGN1 confined fraction is defined as the gray zone between –4 < Log(D) < 1.8. [file Image_1.TIF]

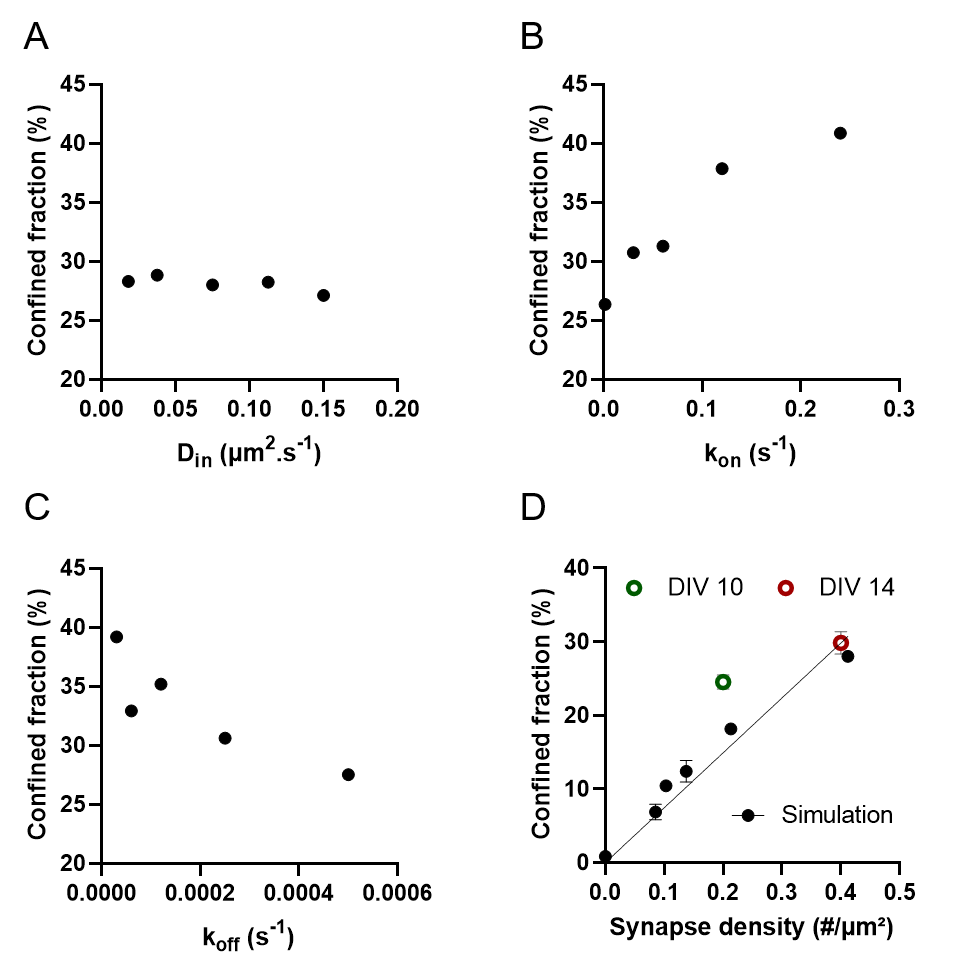

Supplement: Supplementary Figure 2 — Impact of kinetic parameters and synapse density on the simulated NLGN1 confined fraction. (A–D) Plots showing the fraction of confined NLGN1 molecules as a function of the parameters Din, kon, and koff, or synapse density. Values correspond to the population highlighted in gray in Supplementary Figure 1. When not varied, parameters were Dout = 0.15 μm2.s–1, Din = 0.15 μm2.s–1, Dtrap = 0.006 μm2.s–1, kon = 0.0015 s–1, koff = 0.0005 s–1, and synapse density = 0.4 μm–2 (i.e., all PSDs were active). Pcrossing was adjusted so as to maintain a constant theoretical synaptic enrichment (Pcrossing Dout/Din) (1 + kon/koff) = 4. [file Image_2.TIF]

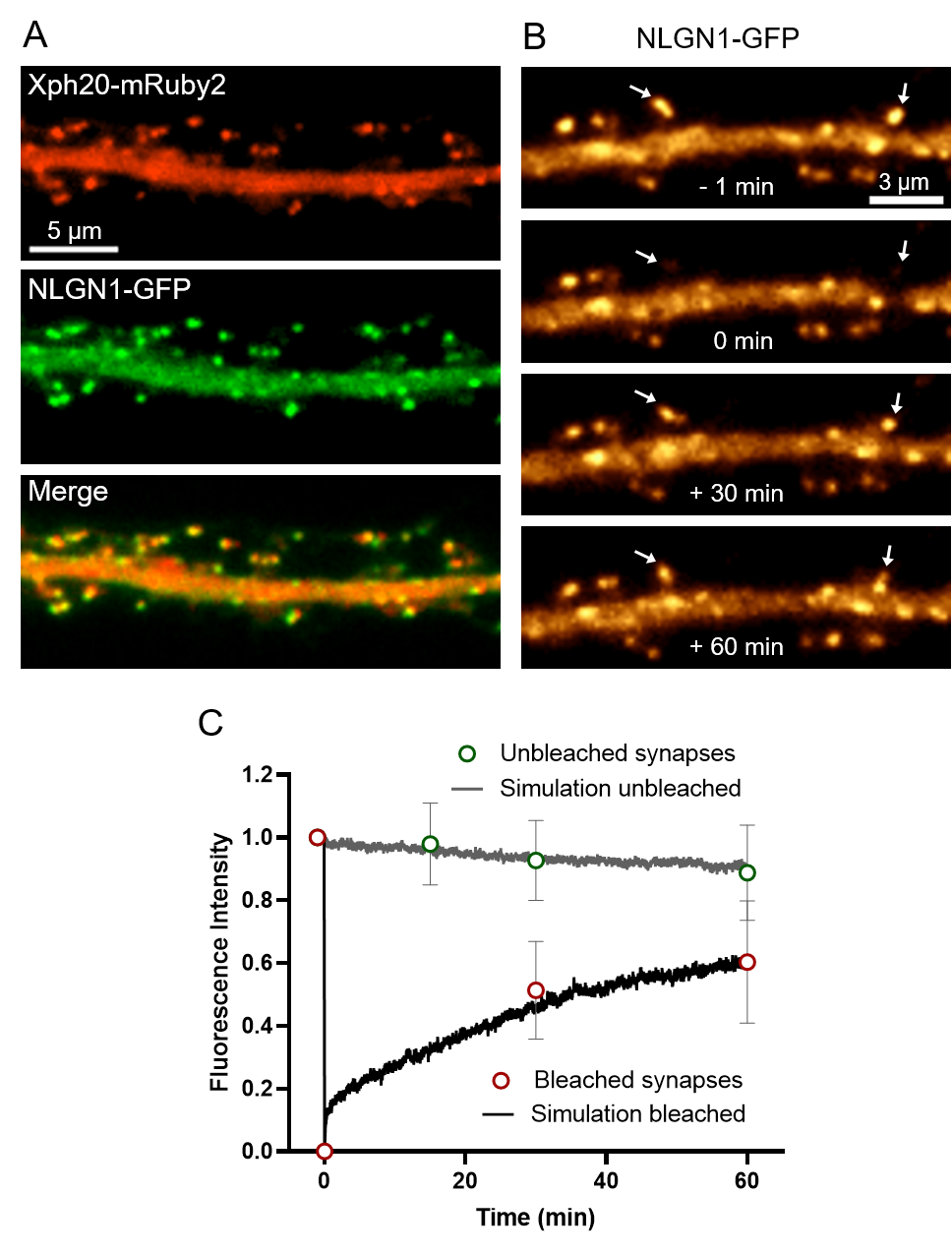

Supplement: Supplementary Figure 3 — Long term FRAP on NLGN1-GFP. (A) Post-synaptic distribution of NLGN1-GFP. Representative images of a dendritic segment from a neuron co-expressing NLGN1-GFP (green) and Xph20-mRuby2 (red), an intrabody to PSD-95. The merged image shows extensive colocalization between the two proteins at post-synapses (yellow signal). (B) Representative 1-hr FRAP sequence performed on a neuron co-expressing NLGN1-GFP and Xph20-mRuby2. The NLGN1-GFP signal was photobleached at time 0 on two post-synapses, and epifluorescence images were acquired after 30 and 60 min. (C) Graph showing the fluorescence intensity of unbleached and photobleached synapses, normalized to the baseline level before photobleaching. Data are mean ± SD for 47 and 54 bleached and unbleached synapses, respectively, out of 9 neurons from 2 different experiments. The curves are computer simulations run for 1 h. The parameter χ2 estimating the goodness of fit calculated for the two experimental points (30 and 60 min) is 0.31 for bleached synapses and 0.014 for unbleached synapses. [file Image_3.TIF]

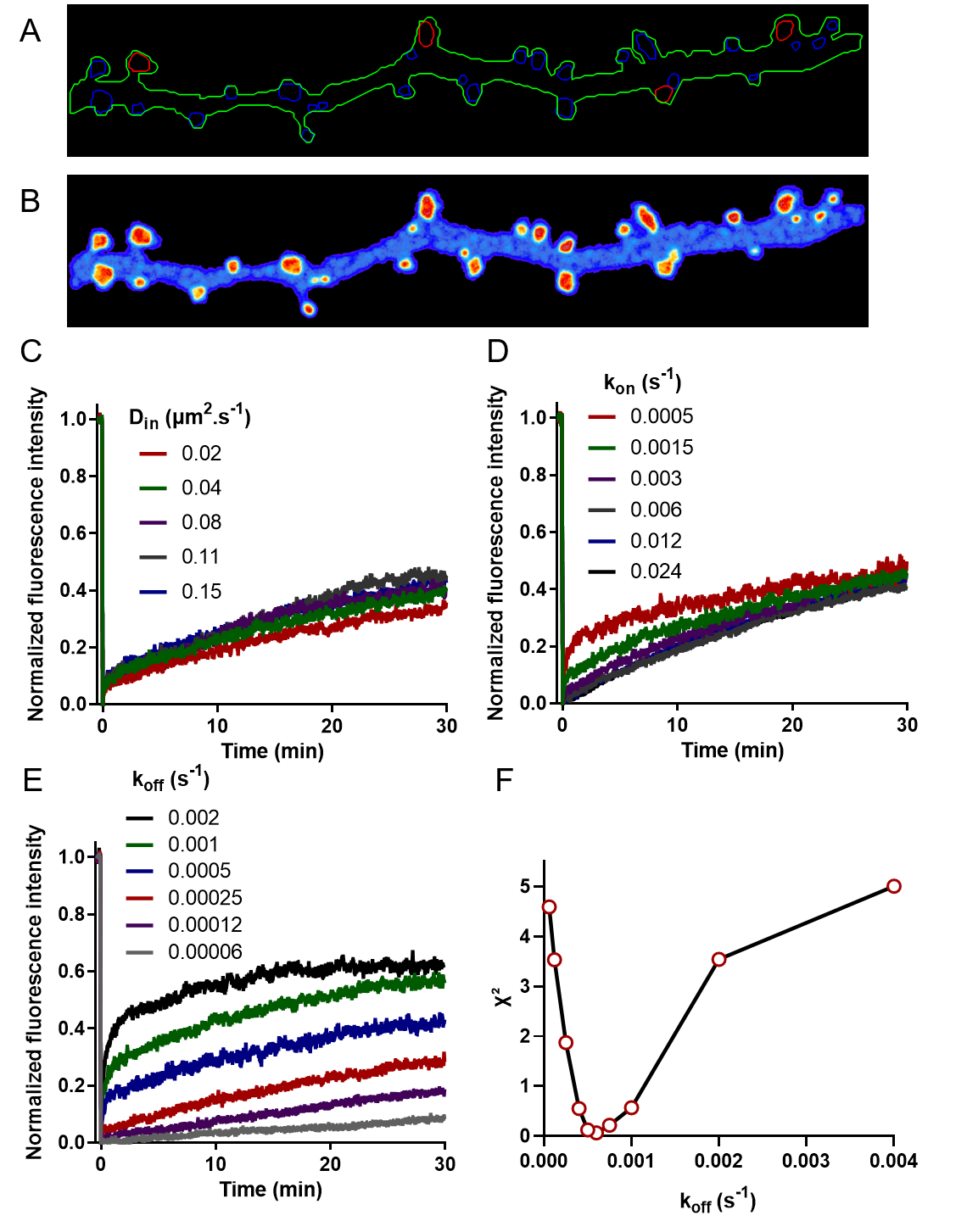

Supplement: Supplementary Figure 4 — Impact of kinetic parameters on simulated FRAP curves. (A) Dendritic geometry used in FluoSim, based on the image of a neuron expressing Homer1c-DsRed. PSDs outlined in red are those which are photobleached at time zero, while PSDs outlined in blue serve as unbleached controls. (B) Heat map of NLGN1 localization with a color code representing the accumulation of NLGN1 in PSDs. (C–E) FRAP curves obtained for individual variations of the parameters Din, kon, and koff, respectively, all other parameters in FluoSim being kept constant. When not varied, parameters were Dout = 0.15 μm2.s–1, Din = 0.15 μm2.s–1, Dtrap = 0.006 μm2.s–1, kon = 0.0015 s–1, koff = 0.0005 s–1, and synapse density = 0.4 μm–2 (i.e., all PSDs were active). Pcrossing was adjusted so as to maintain a constant theoretical synaptic enrichment (Pcrossing Dout/Din) (1 + kon/koff) = 4. (F) Graph showing the coefficient χ2 estimating the goodness of fit, plotted as a function of koff. The minimum of this curve indicates the koff value giving the best fit, which was chosen thereafter in the model. [file Image_4.TIF]

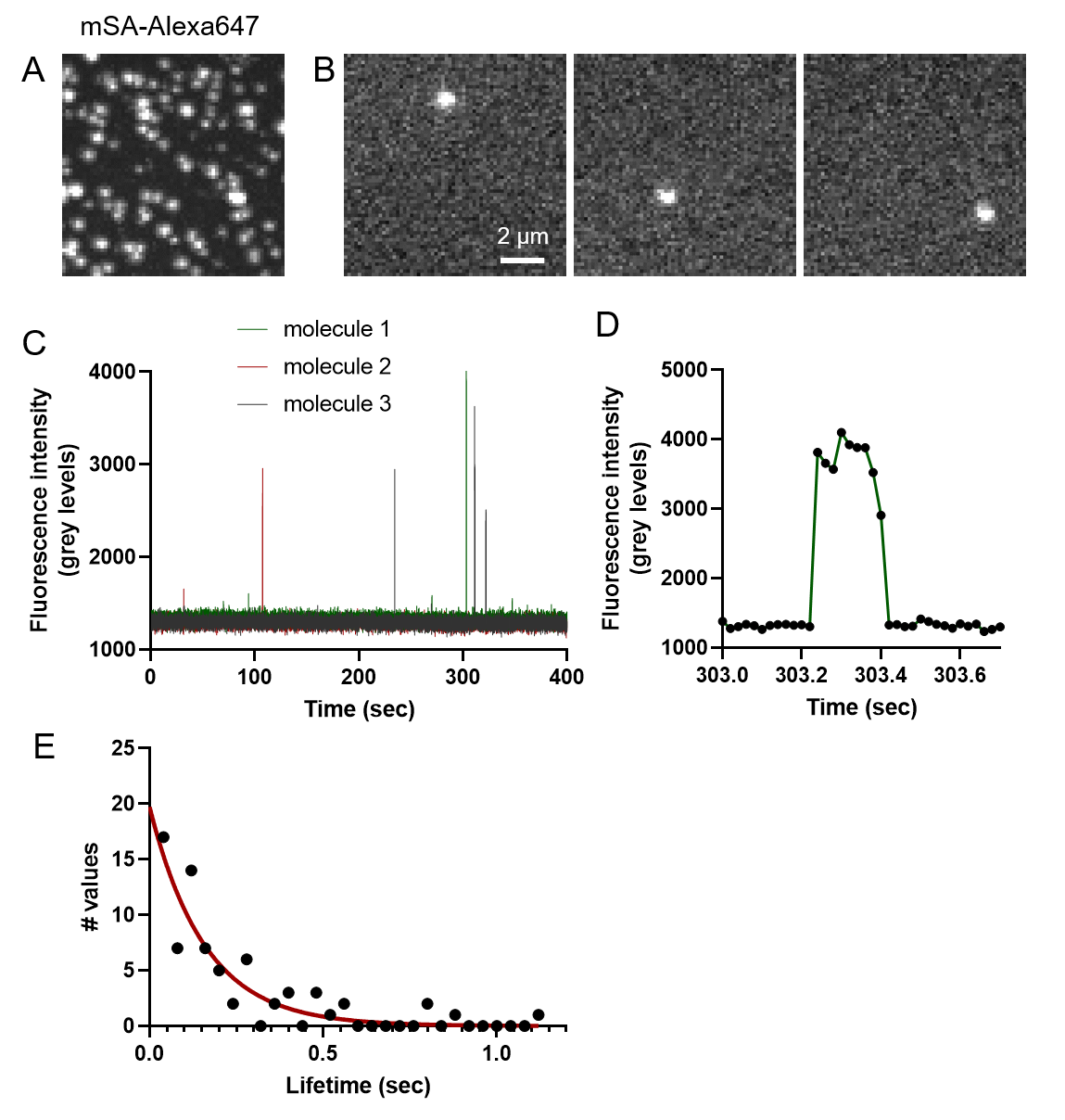

Supplement: Supplementary Figure 5 — Photophysical properties of Alexa647-conjugated mSA in dSTORM conditions. (A) Maximum intensity projection image of a dSTORM sequence run on diluted mSA-Alexa647 molecules (1 nM) immobilized on a glass coverslip. The region of interest is 64 × 64 pixels (10 × 10 μm) and the sequence was 20,000 frames at a 20 ms exposure time per frame (total duration 400 s). (B) Representative single frame images of individual molecules while they emit fluorescence. (C) Fluorescence intensity in gray levels over time for three arbitrary regions of 6 × 6 pixels centered on individual molecules. One or several emission peaks can be clearly distinguished. The frequency of fluorescence emission by individual mSA-Alexa647 molecules, kONFluo, was taken as the average number of peaks divided by the total sequence duration (kONFluo = 0.004 s–1). (D) Zoom on one of the peaks (molecule 1, green), showing the lifetime of the fluorescence emission. (E) Distribution of the fluorescence lifetime calculated from 73 individual emission peaks (black circles). The red curve is a fit with the exponentially decreasing function N0 Exp(–kOFFFluot), where N0 is the number of values at time zero, t is the time in sec, and kOFFFluo = 6.3 s–1 is the characteristic rate constant. [file Image_5.TIF]

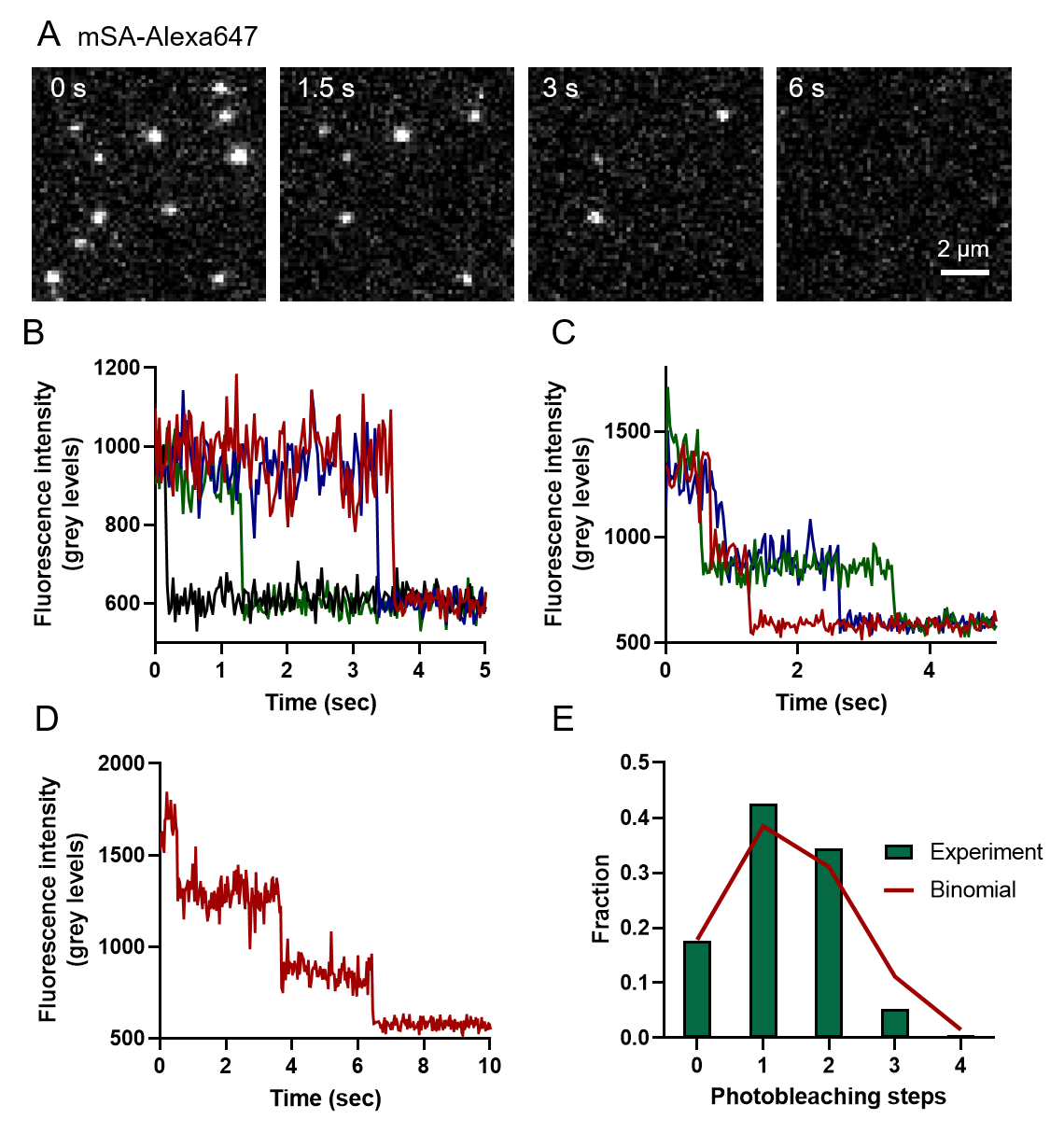

Supplement: Supplementary Figure 6 — Estimation of the number of Alexa647 fluorophores per mSA protein by quantification of photobleaching steps. Alexa-647 conjugated mSA molecules were diluted to 3 nM in Tyrode solution, immobilized on a glass substrate, and imaged by TIRF microscopy at 30 Hz. (A) Representative time lapse images. Note the progressive photobleaching of the mSA molecules. (B–D) Representative fluorescence intensity profiles of individual molecules over time. Each color represents a different molecule. Molecules display mostly 1-step (B) and 2-step (C), but very rarely 3-step (D) photobleaching profiles. (E) Distribution of the number of photobleaching steps computed from 250 individual Alexa647-conjugated mSA molecules. The red curve represents a binomial distribution with 4 binding sites and conjugation probability of Alexa647 to mSA of 0.35. The best fit allows the computation of a 17% fraction of unconjugated mSA, thereby undetectable by fluorescence microscopy. [file Image_6.TIF]

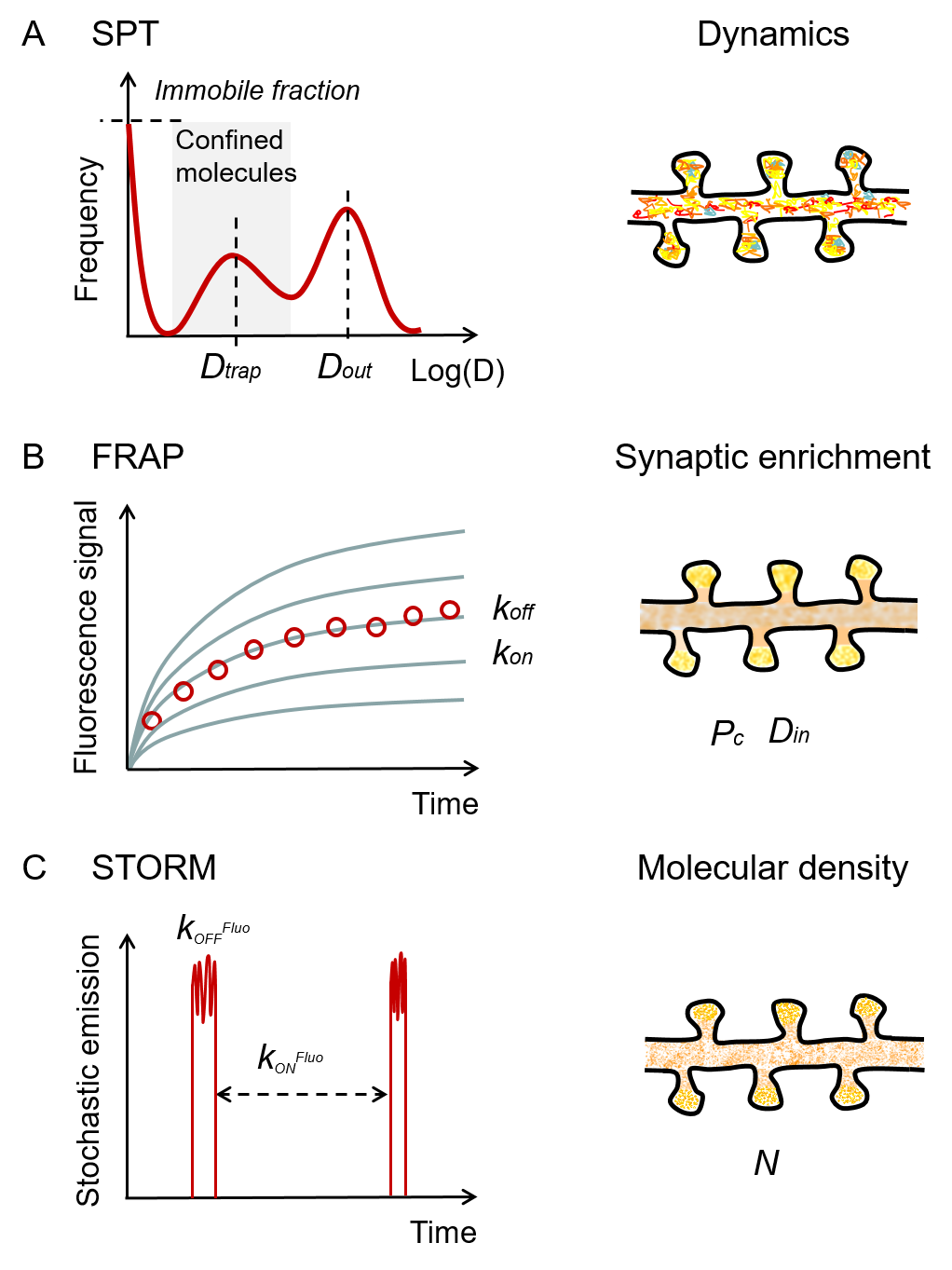

Supplement: Supplementary Figure 7 — General strategy to estimate model parameters entered in FluoSim. (A) Single molecule tracking experiments yield with relatively high precision the NLGN1 immobile fraction, the peak NLGN1 diffusion coefficient outside synapses (Dout), and the peak diffusion coefficient of NLGN1 molecules bound at PSDs (Dtrap). (B) The fit of FRAP experiments with the model allows for the determination of the binding and unbinding rates (kon and koff, respectively) of NLGN1 to the PSD. Further adjustment of the remaining parameters Pc and Din are based on the determination of NLGN1 synaptic enrichment. (C) Quantification of mSA-Alexa647 photophysical rates in dSTORM conditions combined with the other parameters of the model allows for the determination of the copy number of NLGN1 in the dendrite geometry, by matching the absolute number of single molecule detections obtained experimentally. [file Image_7.TIF]
